# Perceptions of climate change across the Canadian forest sector: The key factors of institutional and geographical environment

**DOI:** 10.1371/journal.pone.0197689

**Published:** 2018-06-13

**Authors:** Aitor Ameztegui, Kevin A. Solarik, John R. Parkins, Daniel Houle, Christian Messier, Dominique Gravel

**Affiliations:** 1 Department of Agriculture and Forest Engineering (EAGROF), University of Lleida, Lleida, Spain; 2 Centre d’étude de la forêt (CEF), Département des Sciences Biologiques, Université du Québec à Montréal, Montréal, QC, Canada; 3 Forest Sciences Center of Catalonia (CTFC), Solsona, Spain; 4 Professor, Deparment of Resource Economics and Environmental Sociology, University of Alberta, Edmonton, Canada; 5 Direction de la Recherche Forestière, Forêt Québec, Ministère des Forêts, de la Faune et des Parcs, Québec City, Quebec, Canada; 6 Consortium on Regional Climatology and Adaptation to Climate Change (Ouranos), Montreal, Quebec, Canada; 7 Institut des Sciences de la Forêt Tempérée, Département des Sciences Naturelles, Université du Québec en Outaouais, Ripon, Quebec, Canada; 8 Département de biologie, Université de Sherbrooke, 2500 Boul. de l’université, Sherbrooke, Québec, Canada; Albert-Ludwigs-Universitat Freiburg, GERMANY

## Abstract

Assessing the perception of key stakeholders within the forest sector is critical to evaluating their readiness to engage in adapting to climate change. Here, we report the results of the most comprehensive survey carried out in the Canadian forestry sector to date regarding perceptions of climate change. A total of 1158 individuals, representing a wide range of stakeholders across the five most important forestry provinces in Canada, were asked about climate change, its impact on forest ecosystems, and the suitability of current forest management for addressing future impacts. Overall, we found that respondents were more concerned about climate change than the general population. More than 90% of respondents agreed with the anthropogenic origins of climate change, and > 50% considered it a direct threat to their welfare. Political view was the main driver of general beliefs about the causes of climate change and its future consequences, while the province of origin proved to be the best predictor of perceived current impacts on forest ecosystems and its associated risks; and type of stakeholder was the main driver of perceived need for adaptation. Industrial stakeholders were the most skeptical about the anthropogenic cause(s) of climate change (18% disagreed with this statement, compared to an average of 8% in the other stakeholders), its impacts on forest ecosystems (28% for industry *vs*. 10% for other respondents), and the need for new management practices (18% *vs*. 7%). Although the degree of awareness and the willingness to implement adaptive practices were high even for the most skeptical groups, our study identified priority sectors or areas for action when designing awareness campaigns. We suggest that the design of a strategic framework for implementing climate adaptation within the Canadian forest sector should focus on the relationship between climate change and changes in disturbance regimes, and above all on the economic consequences of these changes, but it should also take into account the positions shown by each of the actors in each province.

## Introduction

Forest ecosystems, as well as the forest sector in Canada, are highly vulnerable to the effects of climate change due to the magnitude of the projected increases in temperature at northern latitudes, the long temporal horizon of forest planning, and the social and economic importance of forests [1]. After years in which climate policy has focused primarily on mitigation, it is now clear that the efforts devoted to reducing global greenhouse emissions have fallen short in preventing climate from continuing to change at such a rapid pace [2]. There is currently a dire need to adapt forest management practices to the predicted impacts of climate change, as acknowledged by researchers, managers, and policy makers, among others [3,4]. Recently, several governmental reports identified a strategic framework for implementing climate adaptation within the Canadian forest sector [1,5–7]. Despite these efforts, implementation of adaptive practices into forest management remains mostly conceptual, while “boots-on-the-ground” adaptation actions in response to future climate change remain scarce [4,8].

The design and implementation of a comprehensive and successful adaptation strategy for the Canadian forest sector requires the participation of the widest possible range of stakeholders throughout the country [9], including scientists, policy makers, corporate leaders, resource managers, and forest practitioners from diverse educational, social, and geographical backgrounds, all of which are likely to differ in their awareness of climate change, opinions, perspectives, and goals [10,11]. Both awareness [12,13] and risk perception of climate change [14] are important predictors of perceived need for change and willingness to undertake adaptive practices. As such, determining the awareness and risk perception among stakeholders and across provinces, where management policies are implemented, is critical to evaluating their readiness to engage in the adaptation process. Moreover, identifying the driving factors influencing perceptions can help minimize any waste of resources due to erroneous assumptions [15], and avoid conflicts among groups when designing and implementing awareness campaigns or adaptation strategies [16,17].

In this study, we present the results of the most comprehensive survey on perceptions about climate change that has been carried out in the Canadian forestry sector to date. Although some recent studies have assessed the perceptions of climate change in the forest sector for a particular agency or territory [15,18], our study covers a much wider geographical area and range of organizations, allowing us to compare for the first time how key institutions and geographical areas within the forest sector influence position on climate change within Canada. Participants were asked about their perceptions on climate change, its impacts on forest ecosystems, and the suitability of current forest management practices to address these impacts.

The aim of the survey was threefold: (1) To obtain as complete a picture as possible of the positioning of the Canadian forestry sector in the face of climate change and the need to adapt current practices. Likewise, we evaluate the changes in this positioning in the last decade, comparing some of our results with those obtained in a previous survey conducted in 2005 [18]. (2) To quantify how the views, beliefs, and perceived need for adaptation vary with institutional environment (i.e., across stakeholder groups: Federal Government, Provincial Government, Academia, Student, Forest Industry, and Non-Industrial—private company), and with geographical environment (i.e. province of respondent); and (3) To identify the factors (individually and collectively) that contribute to explaining the individual perceptions of participants.

Relative to objective 1, the growing media attention that has been given to climate change in recent years [19], and its high presence in public debate, make us foresee a greater awareness of its importance among the forestry sector, with respect to the results obtained a decade ago. We also hypothesize that, beyond individual factors, the geographical environment will strongly influence position on climate change, derived from their different legislation, forest practices, and exposure to impacts. Finally, we expect that one’s institutional position relative to climate change will affect his or her perceived risk [11]. Our study can be used to identify priority sectors or areas for action when designing awareness campaigns, as well as to adapt the message according to the positions shown by each of the actors in each province.

## Background (context)

In recent years, the volume of studies examining the variables that are related to the acceptance of anthropogenic climate change has grown unprecedentedly [20]. Socio-demographic variables such as gender, age, education, or income have all been identified as significant predictors of climate change awareness and risk perception, although recent studies suggest that the importance of these variables pales in comparison to other individual-level predictors such as ideology or political orientation [20,21]. In addition to these factors, the institutional and geographical environment play an important role, although they have been studied much less [11]. The personal opinion of respondents is likely to be affected by their institutional environment (i.e. type of stakeholder: federal government, industry, academia, etc.), which can amplify or attenuate their judgement [22]. Moreover, there can be significant variation in responses depending on the province in which respondents work and, consequently, their exposure to changes in climate, impacts, legislation, and forest practices [23]. For instance, the direct exposure to changes in climate and extreme weather conditions a respondent experiences–which will be highly variable across geographical areas–is known to influence their perceptions on climate change [14,24]. In order to analyse the real effect of the institutional and geographical environment on the perceptions of stakeholders, it is thus necessary to take into account the multiple factors hypothesized to affect climate change perceptions at the individual scale, as well as the potential interactions among these factors [11].

## Methods

### General approach

In this study, we use machine learning algorithms to analyze the combined influence of socio-demographic, geographical, and institutional predictors of climate change perception across the Canadian forest sector. This technique allows a more flexible approach than more traditional regression techniques, while providing robust ranking of variable importance, even among highly correlated predictors [25]. Further, this technique has already been used in social sciences studies to explore the relative influence of several factors in shaping climate change perception and risk awareness [26], but it has rarely been used in this type of survey research.

### Sample

This research was approved by the Institutional Ethics Committee on Research with Human Beings (CIEREH) of the University of Québec à Montreal (Approval Code: S-703000). Written informed consent, was obtained from the participants. During the spring of 2015, data was collected from individuals with a professional interest in the management of Canadian forests. To capture potential differences in opinion across Canada, we contacted individuals from the five provinces where the forest sector has the most economic value, comprising more than 90% of workers in the Canadian forest sector: Québec, British Columbia, Ontario, Alberta, and New Brunswick [27]. We searched online employee directories of federal and provincial agencies with jurisdiction of forest management or forest-related research in each province (i.e., Canadian Forest Service, Provincial Ministries of Forests, etc.) as well as those of academic institutions and research centers related to forest ecology or forest management. We selected all the employees whose job titles suggested that their duties were related to the forest sector, resulting in a database of 3,244 potential participants who were then contacted directly through email. To reach individuals in private companies we contacted each provincial Association of Professional Foresters, as well as the Canadian Forestry Adaptation Community of Practice (FACoP), who distributed the survey among their members or associates. We additionally distributed the survey among students of the same academic faculties identified above, to incorporate opinions of future forest leaders who will inherit climate challenges in the forest sector, and to evaluate if their opinions differed from the other stakeholder groups.

### Survey design

**Fig 1:**
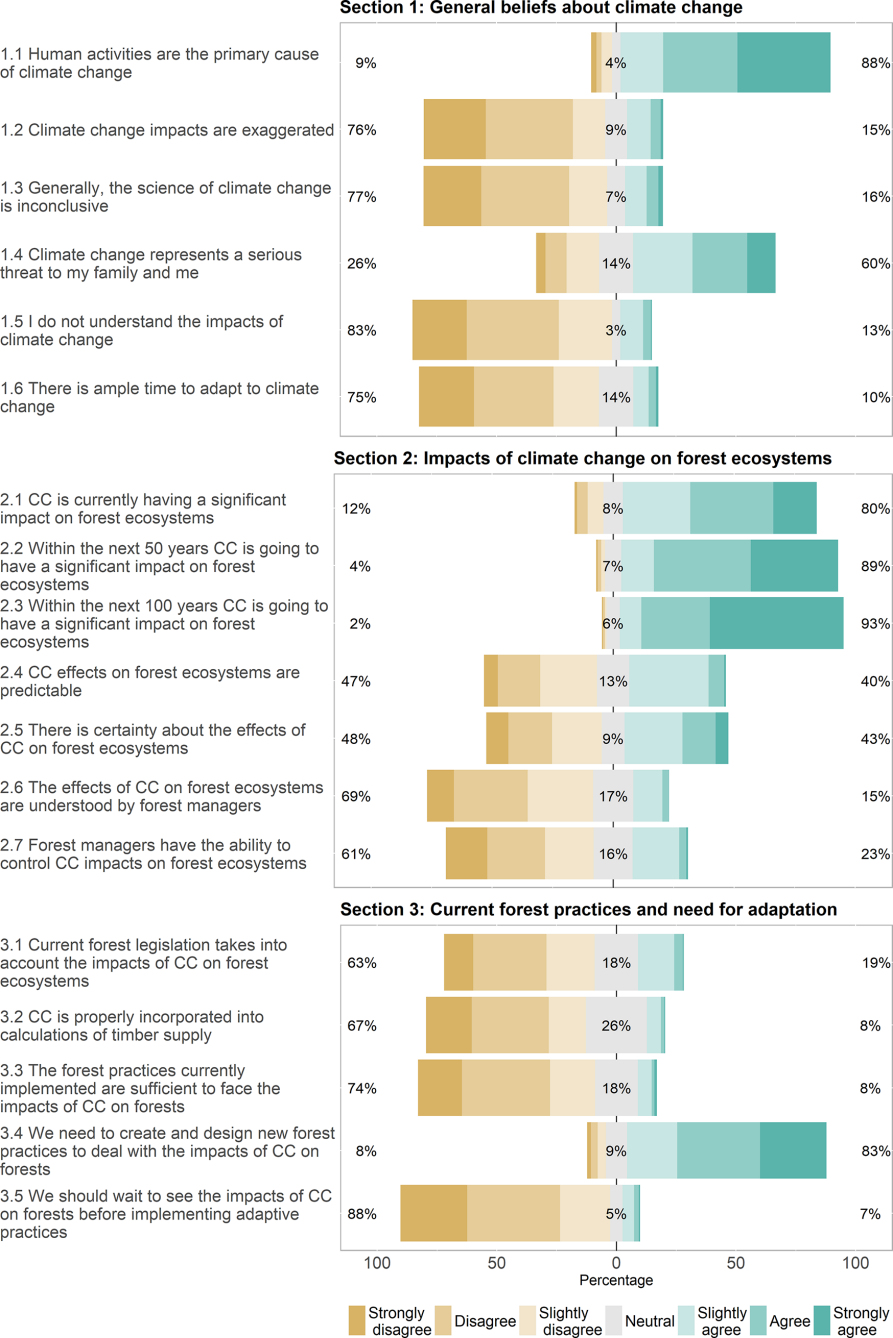
Proportion of overall responses along a 7-grade Likert scale in a survey on perceptions of climate change and its impacts on forest ecosystems across the Canadian forest sector (n = 974). Percentages for each statement represent, from left to right, the amount of disagreements (strongly disagree, disagree, slightly disagree), neutral responses, and agreements (slightly agree, agree, strongly agree).

We used an internet-based survey platform (SurveyGizmo, Boulder, CO, USA) for data collection. The survey was divided into 3 sections: (i) general statements about climate change and its potential impacts (6 statements), (ii) impacts of climate change on forest ecosystems (7 statements), and (iii) current forest management practices and the need to adapt to climate change (5 statements). Respondents specified their degree of agreement with each statement using a 7-grade Likert scale ranging from “strongly disagree” to “strongly agree” (Fig 1). The questions in the first and second sections followed the same phrasing as those included in a smaller survey conducted among forestry experts in 2005 [18], which allowed us to assess the potential temporal changes in perceptions over the past decade.

As predictor variables, we recorded the geographical environment of respondents (province of residence) and their institutional environment (type of stakeholder: Federal Government, Provincial Government, Academia (Professor/Staff), Student, Forest Industry, and Non-Industrial—private company). Additionally, we collected socio-demographic information known to influence the opinions and perceptions of respondents on climate change (gender, age, highest level of education completed, and political view).

### Data analyses

All analyses were performed using R v.3.0.3 [28]. We investigated differences in the distribution of gender, age, level of education and province across stakeholder groups by Chi-square tests and pairwise post-hoc comparisons with Bonferroni correction for multiple testing. When conducting a survey, the sample must be as representative of the population as possible. In this case, we considered our population to be the whole Canadian forest sector and therefore we took as reference the relative sample size of the forestry sector within each province according to the Labour Force Survey: Québec: 33%, British Columbia: 27%, Ontario: 20%, Alberta: 10%, New Brunswick: 6% [27]. Althoug the overall proportion of respondents from each province in our survey was similar to those numbers, it was not consistent across stakeholder groups (i.e. not all provinces were equally represented in all stakeholders, see S 1). Therefore we applied post-stratification weights to correct biases due to overrepresentation of certain groups [29].

We considered Likert scale items as ordinal variables, so we assessed differences in responses across provinces (or geographical origin) and type of stakeholder (or institutional environment) through Kruskal-Wallis non-parametric tests. We used a t-test for independent samples to compare the results of our two first sections to those obtained in 2005 [18], after checking the assumptions about normality in the data.

To identify the main potential predictors of the obtained responses, we used random forests instead of more classical approaches such as hierarchical multiple regression [21]. A random forest is a set of classification or regression trees, where each tree is constructed based on recursive partitioning [30,31]. Random forests have the advantage of handling large numbers of predictor variables, and are able to provide robust ranking of variable importance even among highly correlated predictors [25,30]. Random forests have shown high performance for both classification and regression problems, and have thus been used successfully in many fields, including the analysis of the main variables that determine the responses to surveys on climate change [26].

To avoid overfitting the models, we decided to use a two-step approach. In the first step, we determined the most influential variables for each question in the survey using the *VSURF* package [32]. Random forests provide an importance estimate for each predictor (variable importance, “VI”), and this package implements an algorithm that ranks the variables in an iterative fashion based on their VI, removing the least important ones and only retaining those variables truly related to the response variable [32,33]. In the second step, we used the function *ctree* in the *party* package [25] to construct a single conditional inference tree for each survey statement based on the explanatory variables previously retained in step 1, as suggested by Lee et al. [26]. This function recursively performs univariate splits of the dependent variable based on values of a set of covariates. We fixed the threshold to drop a variable at *p*-value = 0.05. Conditional inference trees allow for any type of response variable, including ordinal and categorical variables, as in our case. We calculated the classification accuracy for each conditional tree, understood as the proportion of correct predictions in a validation subset of the dataset. By conducting this two-step approach we were able to use as much data and predictors as possible since there are no constraints in terms of missing data, and to identify the most important variables without the limitations of more traditional approaches and the risks of overfitting the conditional trees.

## Results

### Overall views on climate change and the need for adaptation in the Canadian forest sector

A total of 1158 individuals started the survey and 974 completed it, resulting in a completion rate of 84%. Moreover, 24.9% of the 3244 individuals that were contacted directly through email responded. However, the overall response rate could not be calculated due to the sampling methodology for private companies. The proportion of respondents by province broadly corresponded to the relative importance of their forest sector according to the Labour Force Survey [27]. (See S1 File for socio-demographic characteristics of respondents).

Overall, we observed widespread acceptance of the existence of climate change and its human origin, as well as its potential impact on forest ecosystems. Only 7% of all respondents expressed some level of disagreement with the anthropogenic origins of climate change, whereas nearly 60% considered climate change as a direct threat to them (Fig 1). While most participants agreed that climate change is currently impacting forest ecosystems, this number increased when given a longer time horizon: approximately 90% of respondents agreed or strongly agreed that the impacts would be significant within the next 50 years. Respondents expressed more doubts in the ability of forest managers to understand and control the impacts of climate change on forest ecosystems (<25% of respondents agreed with those statements; Fig 1). In all provinces of origin, respondents also considered the current legislation and forestry practices to be insufficient to face climate change, and 83% agreed that new forest practices are required (Fig 1).

When comparing these findings with those of a survey conducted a decade earlier among forestry experts [18], there was no significant change in the perceptions about climate change and its impacts in general (Table 1). However, there were significant decreases in the proportion of respondents claiming that they did not understand the impacts of climate change (mean ± SD 3.2 ± 1.6 *(in 2005) vs*. 2.5 ± 1.4 (*in 2015*), *p-value* = 0.002), and that climate science is not conclusive (3.5 ± 1.7 *vs*. 2.6 ± 1.5, *p-value* < 0.001), and an increase in those that consider climate change as a risk for them (3.9 ± 1.6 *vs*. 4.6 ± 1.6, *p-value* = 0.002; Table 1, section 1). A higher proportion of respondents than in 2005 also considered that the impacts of climate change on forest ecosystems are predictable and are understood by forest managers (Table 1, section 2).

**Table 1:** Comparison of scores for respondents in a survey on perceptions of climate change and its impacts on forest ecosystems across Canada between a survey conducted in 2005 [18] and the present study.

Statement		Williamson et al. (2005) (n = 53)	Current study (n = 974)	*p-value*
*Section 1*: *General beliefs about climate change*				
	1.1 Human activities are the primary cause of climate change	5.54 ± 1.69	5.81 ± 1.40	0.250
	1.2 Climate change impacts are exaggerated	2.81 ± 1.40	2.58 ± 1.50	0.243
	1.3 Generally, the science of climate is inconclusive	3.47 ± 1.73	2.63 ± 1.54	**<0.001**
	1.4 Climate change represents a serious threat to my family and me	3.92 ± 1.59	4.63 ± 1.63	**0.002**
	1.5 I do not understand the impacts of climate change	3.22 ± 1.62	2.50 ± 1.36	**0.002**
	1.6 There is ample time to adapt to climate change	2.85 ± 1.45	2.61 ± 1.39	0.249
*Section 2*: *Impacts of climate change on forest ecosystems*				
	2.1 CC is currently having a significant impact on forest ecosystems	5.32 ± 1.37	5.32 ± 1.36	0.990
	2.2 Within the next 50 years CC is going to have a significant impact on forest ecosystems	5.96 ± 1.19	5.95 ± 1.14	0.960
	2.3 Within the next 100 years CC is going to have a significant impact on forest ecosystems	N.A.	6.28 ± 1.05	N.A.
	2.4 CC effects on forest ecosystems are predictable	2.94 ± 1.24	3.72 ± 1.42	**<0.001**
	2.5 There is certainty about the effects of CC on forest ecosystems	3.41 ± 1.50	3.83 ± 1.72	0.053
	2.6 The effects of CC on forest ecosystems are understood by forest managers	2.40 ± 1.02	2.96 ± 1.29	**<0.001**
	2.7 Forest managers have the ability to control CC impacts on forest ecosystems	N.A.	3.08 ± 1.49	N.A.

**Note**: Respondents specified their degree of agreement with each statement using a 7-grade Likert scale ranging from “strongly disagree” to “strongly agree”. Values for the current study are the overall mean ± SD for 974 respondents, and *p -values* indicate significant differences in the mean score for each statement between this study and the survey conducted in 2005 [18], according to a t-test for independent samples. N.A.: results not available.

### Institutional and geographical perceptions: the role of stakeholder type and province

**Fig 2:**
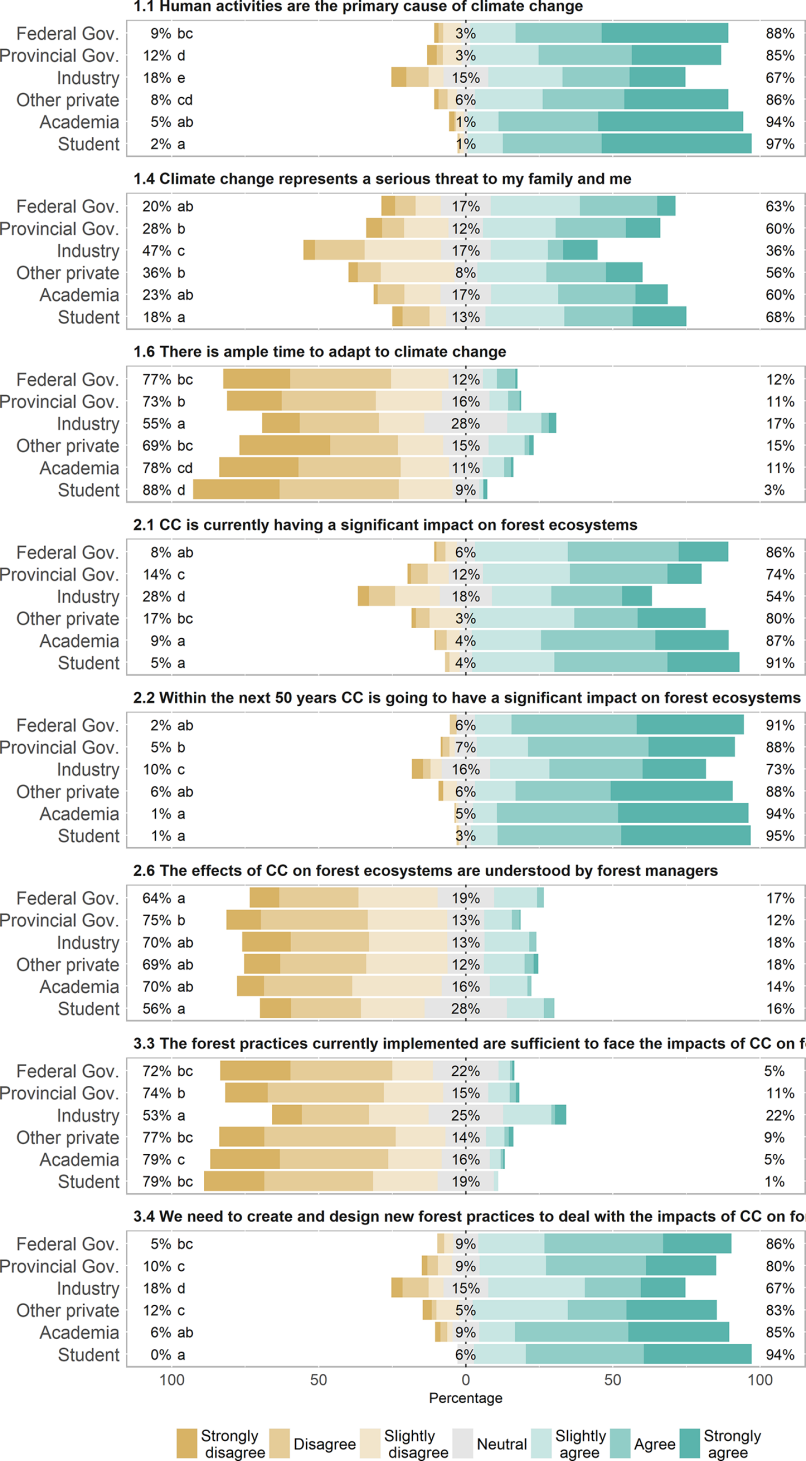
Responses to a survey on perceptions of climate change and its impacts on forest ecosystems by type of stakeholder across the Canadian forest sector (n = 974). The figure includes some of the more relevant results of the survey; the complete results for all the questions are in S2 File. Percentages for each statement represent, from left to right, the amount of disagreements (strongly disagree, disagree, slightly disagree), neutral responses, and agreements (slightly agree, agree, strongly agree). Different letters beside the bars indicate significant differences in the distribution of responses across stakeholder types after a Kruskal-Wallis non-parametric test (n = 974).

Across stakeholder classes, industrial respondents showed the lowest rate of acceptance of human activities as the primary cause of climate change, and were the least concerned about the impacts of climate change on forest ecosystems, both currently and in the future (Fig 2). At the other extreme of the response gradient, academics and students were more convinced of the existence of climate change, more concerned about its effects, and more sensitive to the need for ready response. These two contrasting views on the effects of climate change were also noticeable when respondents were asked about their perceptions of current forest management practices. Respondents from the industry showed lower willingness to adapt, as they considered current practices and associated legislation as already appropriate to face the impacts of climate change: only 53% disagreed with these statements compared to a global average of 76.2% among other stakeholder groups. Accordingly, they were also more confident on the abilities of forest managers to adapt and more reluctant to the need to develop and design new practices to adapt to the challenges associated with climate change (Fig 2 and S2 File).

Compared with the effect of institutional environment (type of stakeholder), the variability in responses across provinces was significantly lower, and was observed mainly in the form of a west-east gradient in some results. More specifically, respondents from the westernmost provinces (either British Columbia or Alberta) tended to be more aware and concerned about the impacts of climate change on forest ecosystems, particularly in the short term, than respondents form Ontario, Québec, or New Brunswick (see S3 File).

### Main drivers of climate change perception

**Fig 3:**
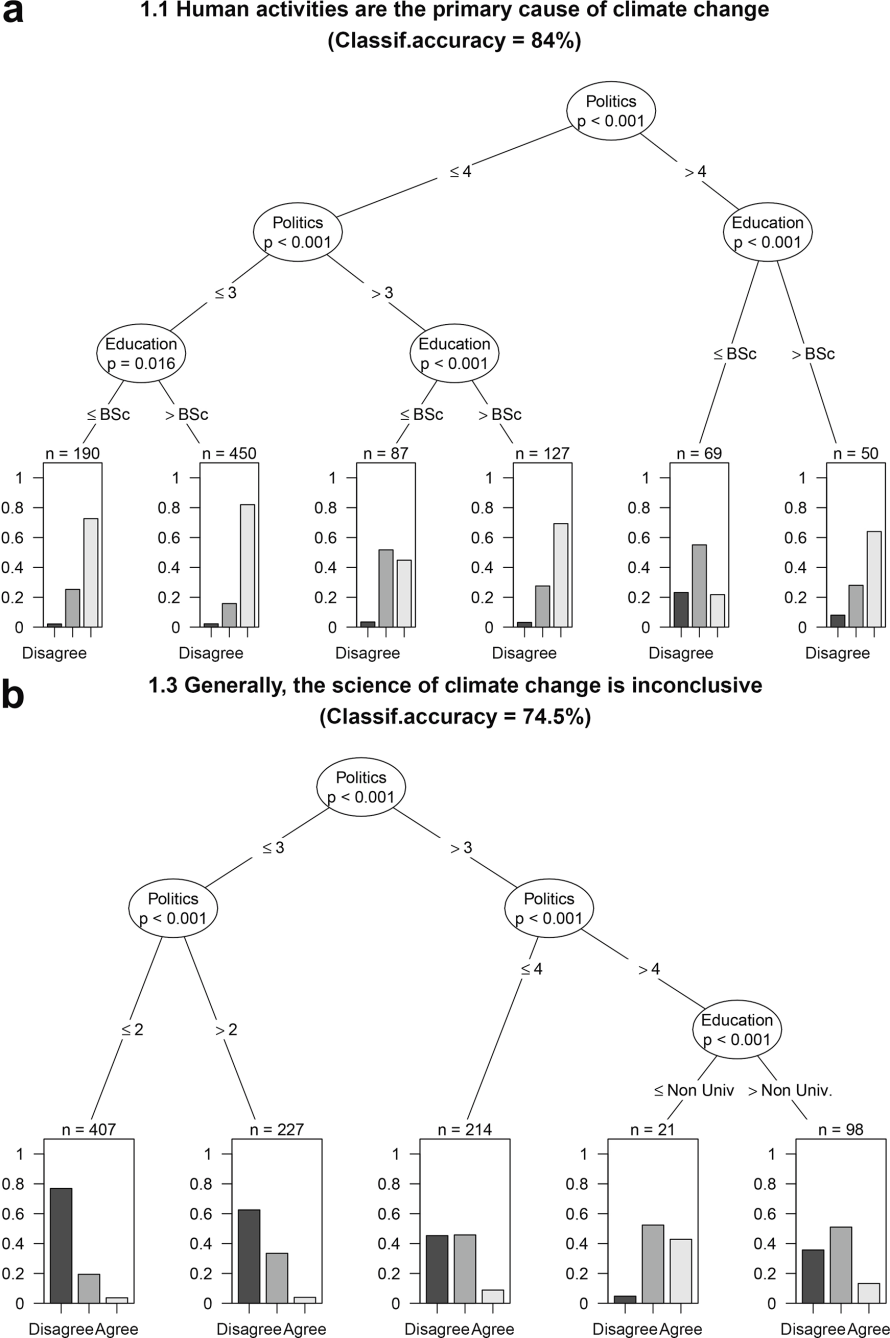
Interaction between politcal view and education level as predictors of perceptions of climate change across the Canadian forest sector. Conditional Inference (CI) classification trees split the dataset into different groups based on certain values of the explanatory variables. At each intermediate mode, dataset is split into two groups based on the values of the explanatory variable indicated in the branches under the node. For statement 1.1, the first node splits the sample into conservatives (political view >4, in a scale from 1 to 7) and liberals, and succesive nodes further split the liberal group into more radical liberals (≤ 3) and moderate liberals (>3). This group is finally split as a function of their educational degree. Those respondents without a Bachelor in Sciences (BSc) are more unsure about the anthropic origin of climate change than those holding a BSc or higher degree. Stacked bar plot at each terminal node indicates the proportion of respondents that disagreed (dark gray), agreed (light gray) or were unsure (gray) about the statement. Total sample size depends on the statement, and can be calculated adding the sample size of the terminal nodes (indicated by brackets). Each tree only shows statistically significant variables at p < 0.05. The classification accuracy for each conditional inference tree is also provided. The CI trees for the all the statements included in the survey can be found at S4 File.

Overall, the random forest models were highly accurate predictors of the observed responses (classification accuracy = 74.0 ± 10.0%, mean ± SD). Political view emerged as the most influential predictor of the individual beliefs about the cause of climate change and its future impacts in general (Table 2, Section 1). Those individuals who identified as “liberals” more strongly supported the anthropogenic origins of climate change (Table 2, Fig 3A), and rejected to a greater extent that climate science was inconclusive (Fig 3B). In addition, they were more likely to see the impacts of climate change as a real problem, not an exaggeration, and believed more strongly that climate change would have an impact on forest ecosystems within the next few decades (Statements 1.2 and 2.2, S4 File).

**Table 2:** Variables selected as potential predictors for each statement, based on variable importance (VI) and using the algorithm developed in the VSURF package. For each statement, only those variables marked with a check mark were used to construct the conditional inference trees shown in Figs 3 and 4 and in S4 File.

Statement			Province	Stake.	Gender	Age	Education	Political View
*Section 1*: *General impacts of climate change*								
	1.1 Human activities are the primary cause of climate change (CC)						√	√
	1.2 CC impacts are exaggerated						√	√
	1.3 Generally, the science of CC is inconclusive						√	√
	1.4 CC represents a serious threat to my family and me		√				√	√
	1.5 I do not understand the impacts of CC			√			√	
	1.6 There is ample time to adapt to CC				√			√
*Section2*: *Impacts of climate change on forest ecosystems*								
		2.1 CC is currently having a significant impact on forest ecosystems	√			√	√	
		2.2 Within the next 50 years CC is going to have a significant impact on forest ecosystems					√	√
		2.3 Within the next 100 years CC is going to have a significant impact on forest ecosystems					√	√
		2.4 CC effects on forest ecosystems are predictable		√				
		2.5 There is certainty about the effects of CC on forest ecosystems	√	√				
		2.6 The effects of CC on forest ecosystems are understood by forest managers	√	√				
		2.7 Forest managers have the ability to control CC impacts on forest ecosystems	√			√		
*Section 3*: *Current practices and need for adaptation*								
		3.1 Current forest legislation takes into account the impacts of CC on forest ecosystems	√	√				
		3.2 CC is properly incorporated into calculations of timber supply		√		√		
		3.3 The forest practices currently implemented are sufficient to face the impacts of CC		√	√	√	√	√
		3.4 We need to create and design new forest practices to deal with the impacts of CC		√				
		3.5 We should wait to see the impacts of CC on forests before implementing adaptive practices						√

**Fig 4:**
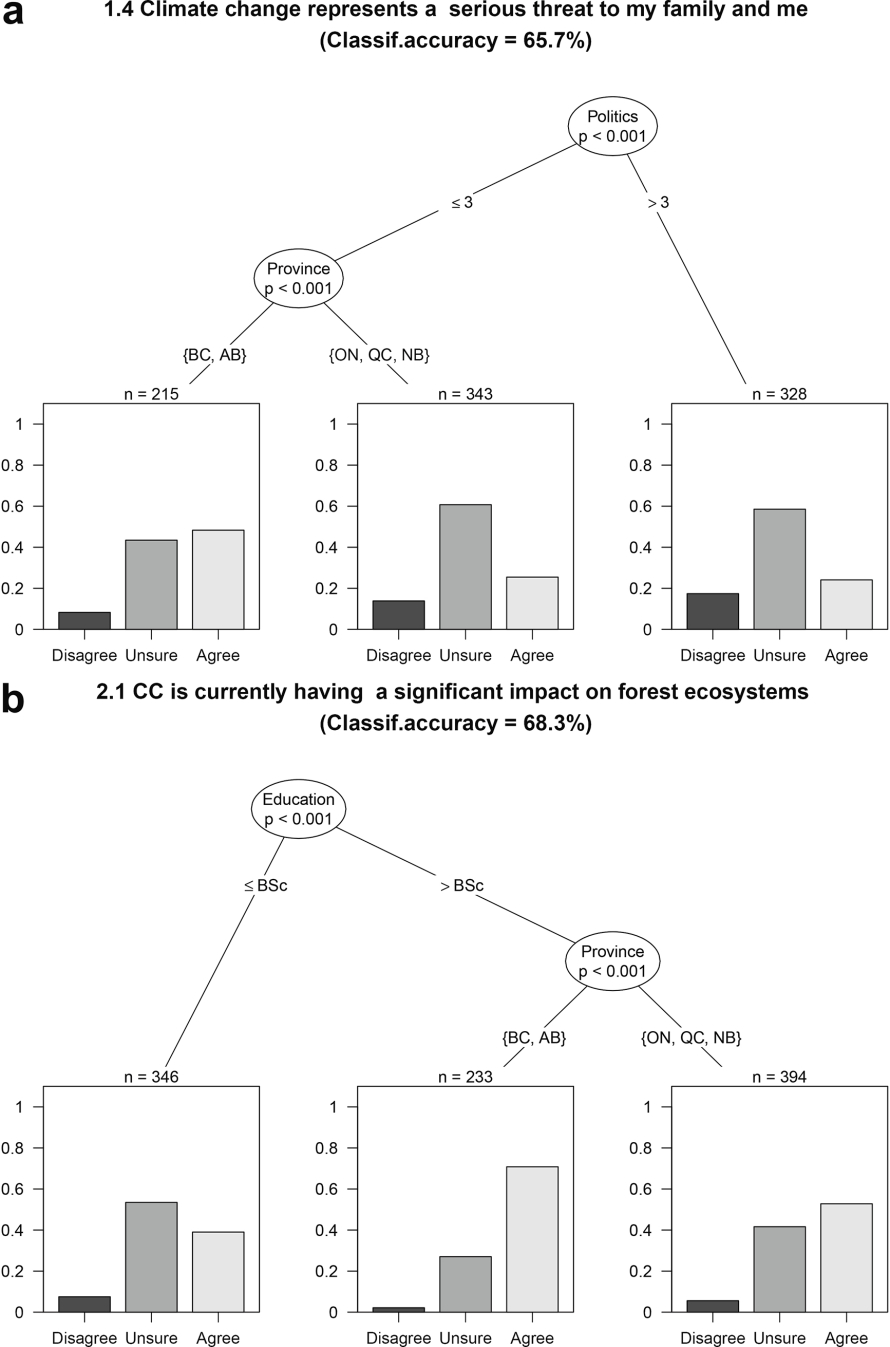
Effect of province of origin, education, and political view as predictors of perceptions on impacts of climate change across the Canadian forest sector. Conditional Inference (CI) classification trees split the dataset into different groups based on certain values of the explanatory variables. At each intermediate mode, dataset is split into two groups based on the values of the explanatory variable indicated in the branches under the node. For statement 1.4, the first node splits the sample into liberals (political view (≤ 3, in a scale from 1 to 7) and moderates and conservatives. Liberal respondents are further split as a function of their province of orgin. Those respondents from westernmost provinces (British Columbia, BC; and Alberta, AB) were more likely than those from Ontario (ON), Québec (QC) or New Brunswick (NB) to consider climate change as a threat to them (a) and already having a significant impact on forest ecosystems (b). In the latter, this distinction was only observed among respondents holding a Bachelor in Sciences (BSc) or a higher degree, whereas those with lower degrees were the most unsure about the impacts on forest ecosystems. Stacked bar plot at each terminal node indicates the proportion of respondents that disagreed (dark gray), agreed (light gray) or were unsure (gray) about the statement. Total sample size depends on the statement, and can be calculated adding the sample size of the terminal nodes (indicated by brackets). Each tree only shows statistically significant variables at p < 0.05. The classification accuracy for each conditional inference tree is also provided. The CI trees for the all the statements included in the survey can be found at S4 File.

Conversely, the province where the respondent resided proved to be the best predictor of the individual beliefs about the impacts of climate change on forest ecosystems and its perception as a threat (Table 2, Section 2; Fig 4). Those respondents from the westernmost provinces (British Columbia and Alberta) were more likely than those from Ontario, Québec or New Brunswick to consider climate change as a threat to them (Fig 4A), and as already having a significant impact on forest ecosystems (Fig 4B).

Finally, the type of stakeholder was the best predictor for statements associated with current forest practices and need for adaptation (Table 2, Section 3). Respondents from private companies were the least satisfied with the current legislation as a means of facing climate change (statement 3.1, S4 File), but paradoxically they also felt there was less of a need for new forest practices (statement 3.4).

The highest level of education attained by respondents interacted with some of the other predictors, particularly with political view, and these complex interactions were captured by our conditional inference trees. For instance, the doubts about the origin and the effects of climate change that were expressed by the most conservative respondents were larger for lower levels of education (Fig 3 and others in S4 File). Moreover, those respondents without a bachelor’s degree were more likely to declare that they did not understand the impacts of climate change, particularly when they were working for the forest industry or a provincial Government (Statement 1.5 in S4 File).

## Discussion

### Views on climate change in the Canadian forest sector, and changes between 2005 and 2015

Overall, respondents showed a high level of awareness of climate change as a risk, and a wide acceptance of its anthropogenic origins. Based on our results, the Canadian forest sector shows a greater consensus (almost 90%) on the cause of climate change than the general public in Canada (40.0%) [34,35]. This high acceptance of the reality and causes of climate change has already been pointed out by previous smaller-scale studies in some Canadian provinces [15,18,36], but our study highlights that it applies throughout the territory. The forest sector has similarly shown more awareness of climate change than the general population in several European countries [13,37–39], likely due to the direct impact of climate and its associated disturbances on the source of their economic well-being.

Interestingly and contrary to what we expected, we found no differences in the perception of climate change causes and impacts on forest ecosystems with respect to a similar survey conducted a decade earlier with 53 forest managers [18]. Although any direct comparison between these surveys should be made with caution, this result is striking given the high media coverage of climate change issues since 2004 [19]. It does highlight that the awareness of climate change and its associated impacts/issues may have reached a plateau in the Canadian forest sector over the last decade, making it difficult to further raise awareness, probably due to other underlying factors.

### Predictor variables of climate change perception: The role of provinces and type of stakeholder

Previous studies surveying the Canadian general population identified substantial differences in opinion at the regional level: respondents within provinces contributing the most to CO_2_ emissions (Alberta and Saskatchewan) believed the least that humans are the cause of climate change [34,35,40]. Although we expected a similar variation in our survey on the forest sector, we did not find such a marked trend, and the views on the existence of climate change and its causes were consistently similar across the studied provinces (see S3 File). In contrast, the province of origin was the best predictor of perceptions about the impacts of climate change on forests and the risk that this currently poses. Although the underlying causes of these differences between provinces can be varied and complex, we suggest that this is likely due to the direct exposure to climate change related extreme disturbance events [14,24,41] rather than different provincial legislation or culture. Previous studies have found that most forest sector employees have experienced the effects of climate change mainly through disturbances and extreme events [37–39]. For example, an unprecedented outbreak of the mountain pine beetle in the 1990s and 2000s affected over 18.1 million hectares in British Columbia and Alberta, and has been directly linked to climate change [42]. Fire activity in the western boreal forests has also increased in recent years, and and some recent catastrophic wildfires have also been linked to climate change [43–45]. These phenomena have strongly impacted the forest sector in these provinces and have undoubtedly contributed to an increased perception of the associated risks. However, it is not possible to state unequivocally the causes behind these geographical patterns.

Across stakeholders, the greatest skepticism about climate change and its consequences was observed among the industry's respondents, who often took positions far distant from other groups, especially academics and students. In fact, stakeholder group was the best predictor for the perceived need for adapting forestry practices. This is the first time such a difference among stakeholders is observed for the forest sector, although differences in risk perception between industry and the university environment were already observed in 2004 by a study surveying policy makers (in general) in Canada [11]. However, it is noteworthy that this study obtained similar responses for industry and government, both in terms of risk perception and ecological and economic beliefs, while in our study government respondents, both provincial and federal, show intermediate positions between the two extremes represented by industry and students. This could indicate an evolution in opinions among government workers, suggesting that government initiatives in this regard may have yielded positive results [5,7,46].

The importance of political view as predictor of climate change perceptions has already been observed for the general population and policy makers of the United States and Canada, where the largest differences in views of the existence of global warming appear across partisan affiliation [11,20,26,40]. For many questions, education interacted with political views, where a higher level of education usually corresponded with a much higher risk perception, especially for respondents who identified as “liberal”, as reported for the United States [47].

### Policy implications

The high overall awareness about climate change and its causes and impacts observed in this study could indicate that campaigns targeting the forest sector in general are no longer needed. In addition, and considering the lack of changes in the acceptance of climate change over the last 10 years, we can conclude that recent awareness campaigns are not succeeding, and they should be redesigned if they intend to reach those few pockets that still remain skeptical. Nevertheless, a greater proportion of respondents–as compared to the 2005 survey [18]–declared that they understood the impacts of climate change and they trusted the ability of scientists to predict impacts on forest ecosystems and of forest managers to understand them and adapt management accordingly. There was also a significant increase in the perception of climate change as a risk for forests. These results are encouraging from a policy point of view since the inability to predict impacts with sufficient detail and certainty (i.e. uncertainty aversion) has been commonly identified as one of the main barriers to implementation of adaptive practices [15,48,49].

At first sight, our results suggest that investing in education may seem an effective tool to increase awareness of climate change. However, McCright and Dunlap [47] reported that the level of education in the United States is correlated with greater climate change risk perceptions only among liberals, but with a lower risk perception for conservatives, suggesting that higher education ultimately strengthens ideological views. This change in the sense of the interaction between education level and political view has not been observed for European forest owners [50], and we could not detect it in our dataset either, but attention should be paid to this possibility when designing awareness-raising campaigns.

Our results also suggest that plans to promote adaptation should be designed to take into account the specificities of forest industry workers. The differences in responses for the industry can be partly explained by their different socio-demographic characteristics or political view—they were on average more conservative than the other groups–but we were able to detect an intrinsic effect attributable to the industrial sector itself, beyond the individual characteristics of the respondents. Industrial respondents are likely to be more concerned about other issues (economics) that can compete with climate change challenges, and to be more aware of the costs of implementing adaptive measures [11]. These issues should be explicitly taken into account in the design of future climate change adaptation campaigns or strategies for the forest sector.

Finally, the provinces most exposed to forest disturbances linked to climate change were more aware of its impacts and risks, highlighting that such events can increase concern about climate change [19]. However, the industrial sector remained the most skeptical even in these provinces, and while recognizing the impacts of climate change on forests, it is still reluctant to see it as a risk or threat to its activity and livelihood. Recent research has shown that the analysis of the role of risk and uncertainty aversion on forest adaptation decision is still an open question [49]. In this sense, the communication strategy should focus on highlighting the relationship between climate change and the current main forest disturbances, with particular emphasis on the economic consequences for the forest industry and the threat it poses to its activity and jobs.

## Conclusions

Through this survey, we were able to take the most complete picture to date of the positioning of the Canadian forestry sector with respect to climate change, its impacts, and the willingness to undertake adaptation measures. The degree of awareness and the willingness to adapt were high even for the most skeptical groups, although they remained relatively unchanged based on a survey made eleven years earlier.

Both the institutional environment (type of stakeholder) and geographical origin (province) were found to be important predictors beyond the individual ideology. Westernmost provinces proved to be more worried about the current impacts of climate change, whereas the industry sector was the most skeptical about the anthropogenic origin of climate change and the need for new management practices. We suggest that the design of a strategic framework for implementing climate adaptation within the Canadian forest sector must take into account these specificities, focusing on the relationship between climate change and changes in disturbance regimes, and above all on the economic consequences of these changes. Considering the willingness to adapt is high throughout the sector, identifying the main barriers that prevent implementing on-the-ground adaptations to management practices across stakeholders is critical, and should be the aim of future research in this field.

## Supporting information

S1 File Socio-demographic information of respondents. Distribution by gender, age, education level, province, stakeholder and political view of respondents in a survey on perceptions of climate change in the Canadian forest sector.
(PDF)

S2 File Survey responses by type of stakeholder. Responses by type of stakeholder for the first three sections of a survey on perceptions of climate change in the Canadian forest sector.
(PDF)

S3 File Survey responses by province of respondent. Responses by province of origin for the first three sections of a survey on perceptions of climate change in the Canadian forest sector.
(PDF)

S4 File Conditional inference trees. Conditional Inference (CI) classification trees for predicting perceptions of climate change and its impacts on forest ecosystems across the Canadian forest sector.
(PDF)
