# Supplementary material for: Perceptions of climate change across the Canadian forest sector: The key factors of institutional and geographical environment
Source: PLoS One. 2018 Jun 13;13(6):e0197689. doi: 10.1371/journal.pone.0197689 (PMC5999070; doi:10.1371/journal.pone.0197689)
Supplement: S1 File — Distribution by gender, age, education level, province, stakeholder and political view of respondents in a survey on perceptions of climate change in the Canadian forest sector. (PDF) [file pone.0197689.s001.pdf]

Supplementary Material S1
Socio-demographic information of respondents

The most represented province was Québec, with 332 respondents (34.1%), followed by Ontario (n = 204, 20.9%), British Columbia (n = 195, 20%), and Alberta (n = 165, 16.9%), while New Brunswick was the least represented (n =78, 8%). The proportion of respondents by province broadly corresponded to the relative importance of their forest sector, according to the Labour Force Survey: Québec: 33%, British Columbia: 27%, Ontario: 20%, Alberta: 10%, New Brunswick: 6% (Canadian Forest Service 2014). Almost half the respondents worked for either the Provincial (n = 335, 34.4 %) or the Federal Government (n = 131, 13.4%). Academic researchers and students accounted for 22.7% and 14.7% of the total number of respondents, respectively, whereas 14.8% of the respondents (n = 144) reported to work for the private sector (79 for forest industries and 65 for consulting or other private enterprises).

All stakeholders had similar age and gender structure, with the exception of students, where there was a greater proportion of young individuals and also a greater proportion of females (55.7% vs. 26.2% for all other groups, Figure S1.1 & Figure S1.2). Moreover, there were also significant differences among stakeholders in the distribution of the educational level (Figure S1.3). The number of individuals with a doctorate was higher for Academia and the Federal Government than for the other groups, whereas the proportion of individuals who have not received university education was highest in the industrial sector.

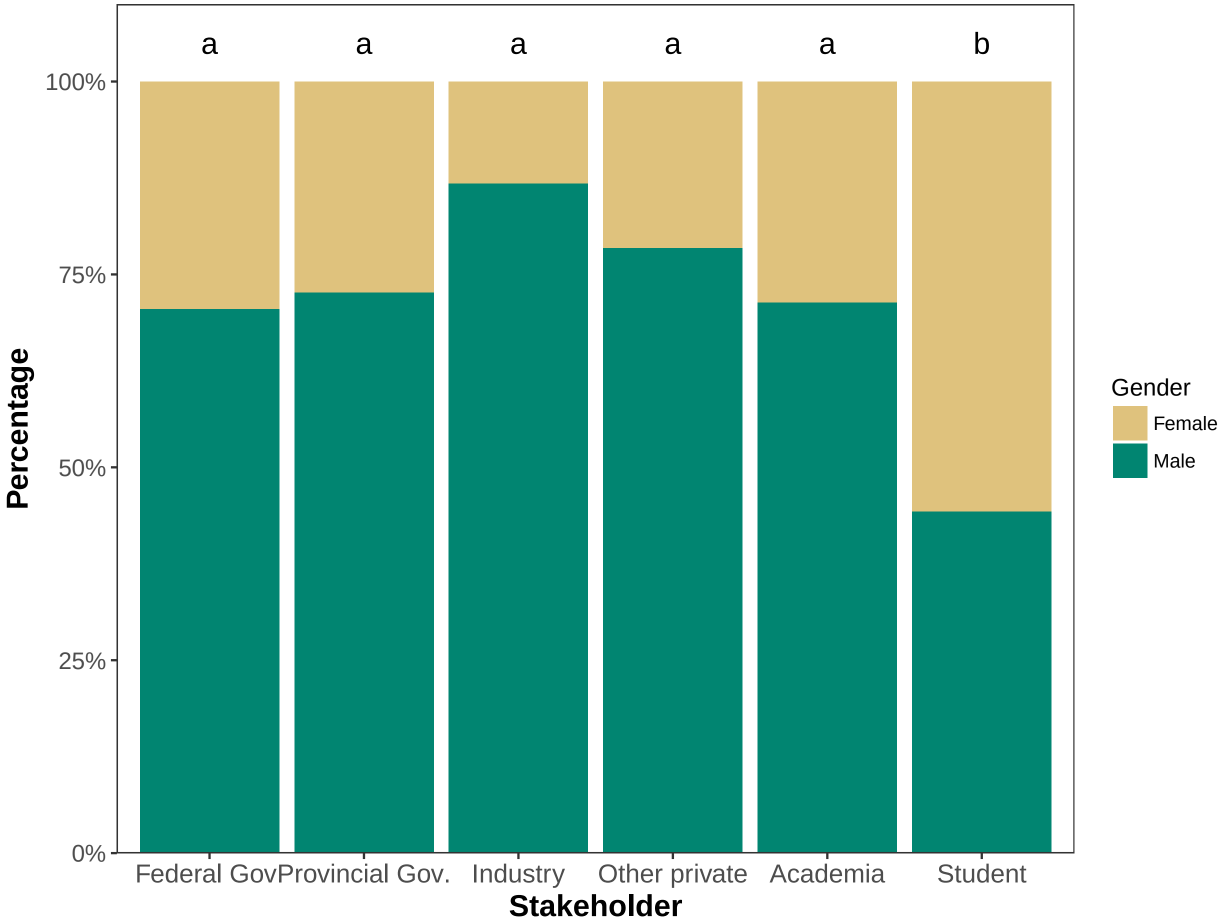

Figure S1.1. Distribution by gender and stakeholder for respondents a survey on perceptions of climate change and its impacts on forest ecosystems in Canada. Different letters above bars indicate significant differences in gender distribution across stakeholders, according to Chi-square statistics after Bonferroni correction for multiple comparisons (n= 974).

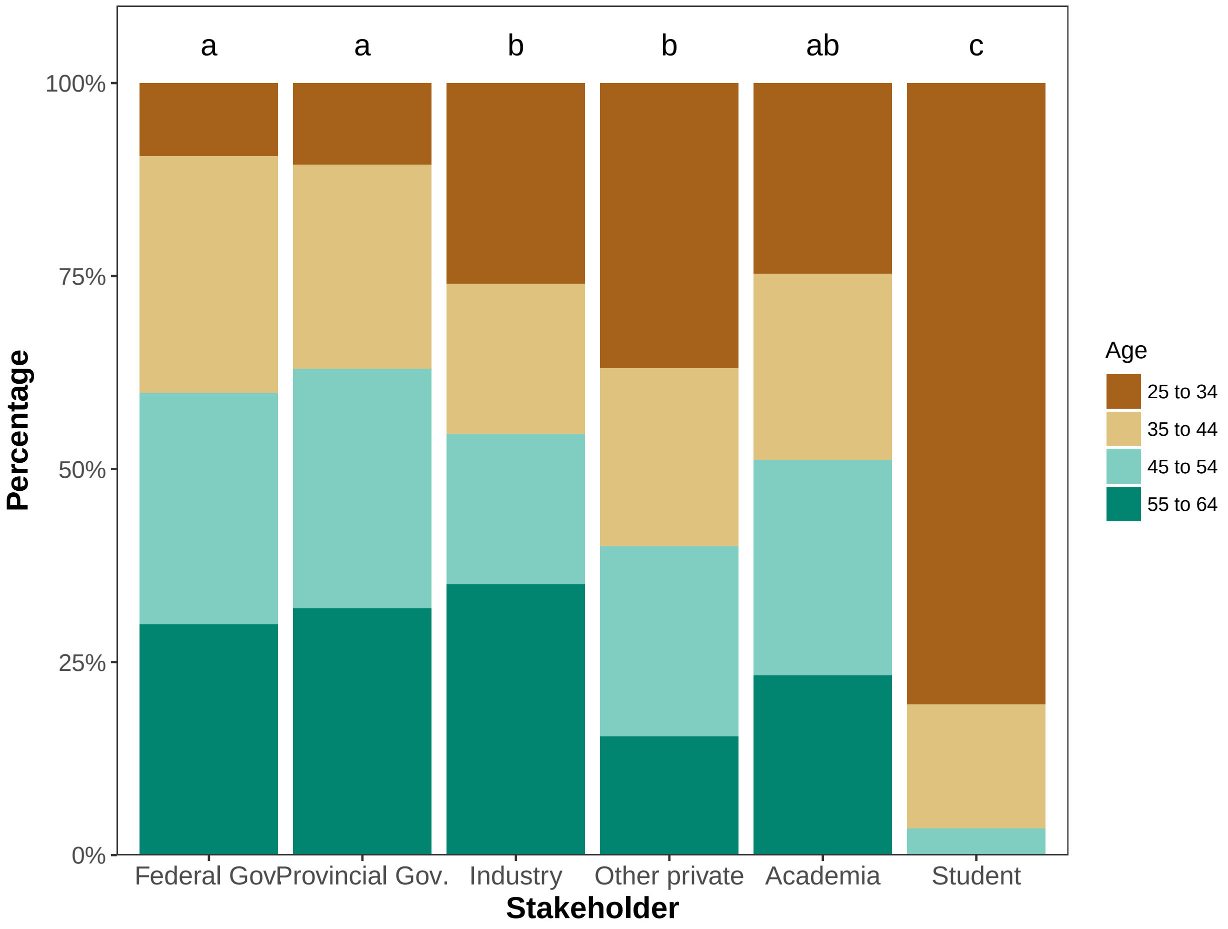

Figure S1.2. Distribution by age and stakeholder for respondents in a survey on perceptions of climate change and its impacts on forest ecosystems in Canada. Different letters above bars indicate significant differences in age distribution across stakeholders, according to Chi-square statistics after Bonferroni correction for multiple comparisons (n= 974).

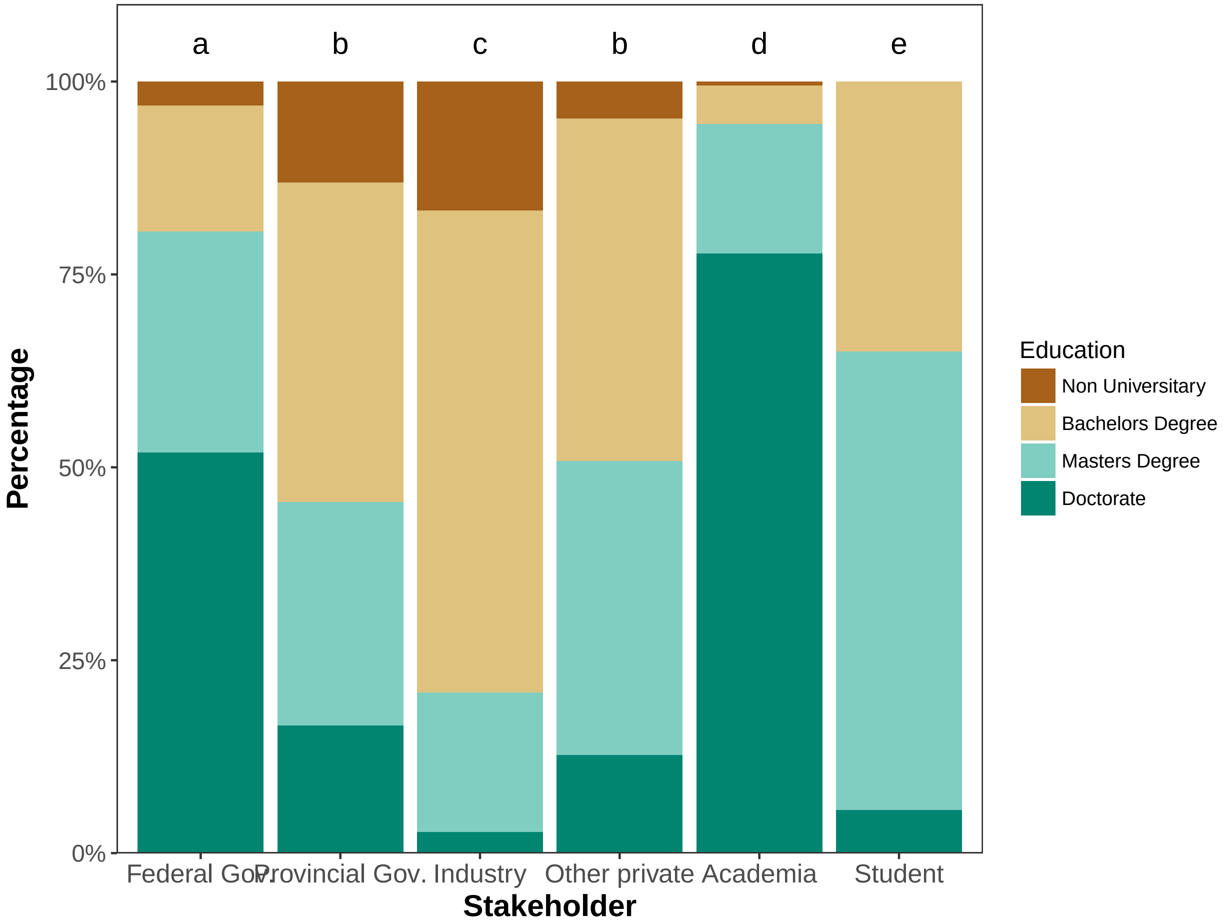

Figure S1.3. Distribution by maximum education level attained and stakeholder for respondents in a survey on perceptions of climate change and its impacts on forest ecosystems in Canada. Different letters above bars indicate significant differences in distribution of education levels across stakeholders, according to Chi-square statistics after Bonferroni correction for multiple comparisons (n= 974).

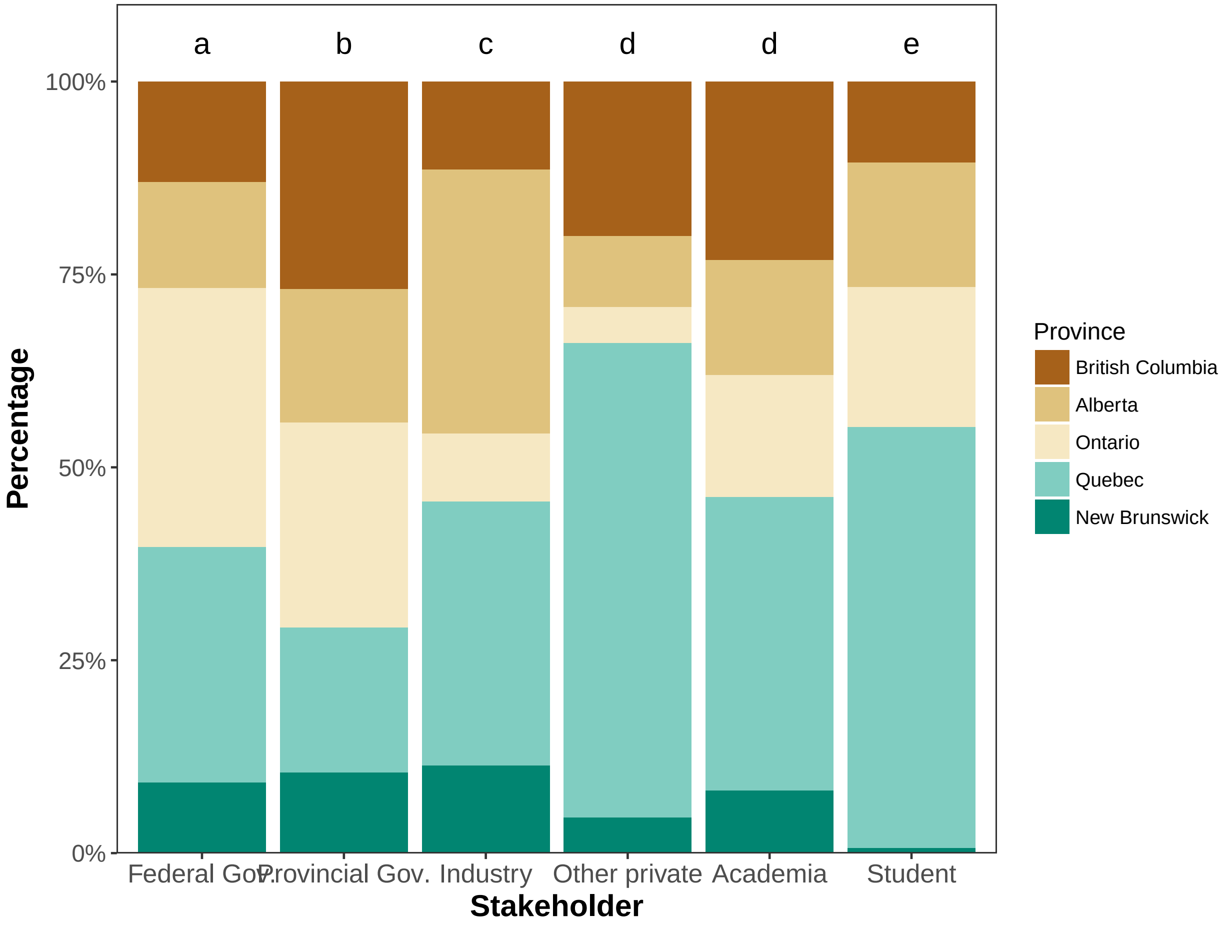

Figure S1.4. Distribution by Province and stakeholder for respondents in a survey on perceptions of climate change and its impacts on forest ecosystems in Canada. Different letters above bars indicate significant differencesin distribution of provinces across stakeholders, according to Chi-square statistics after Bonferroni correction for multiple comparisons (n= 974).

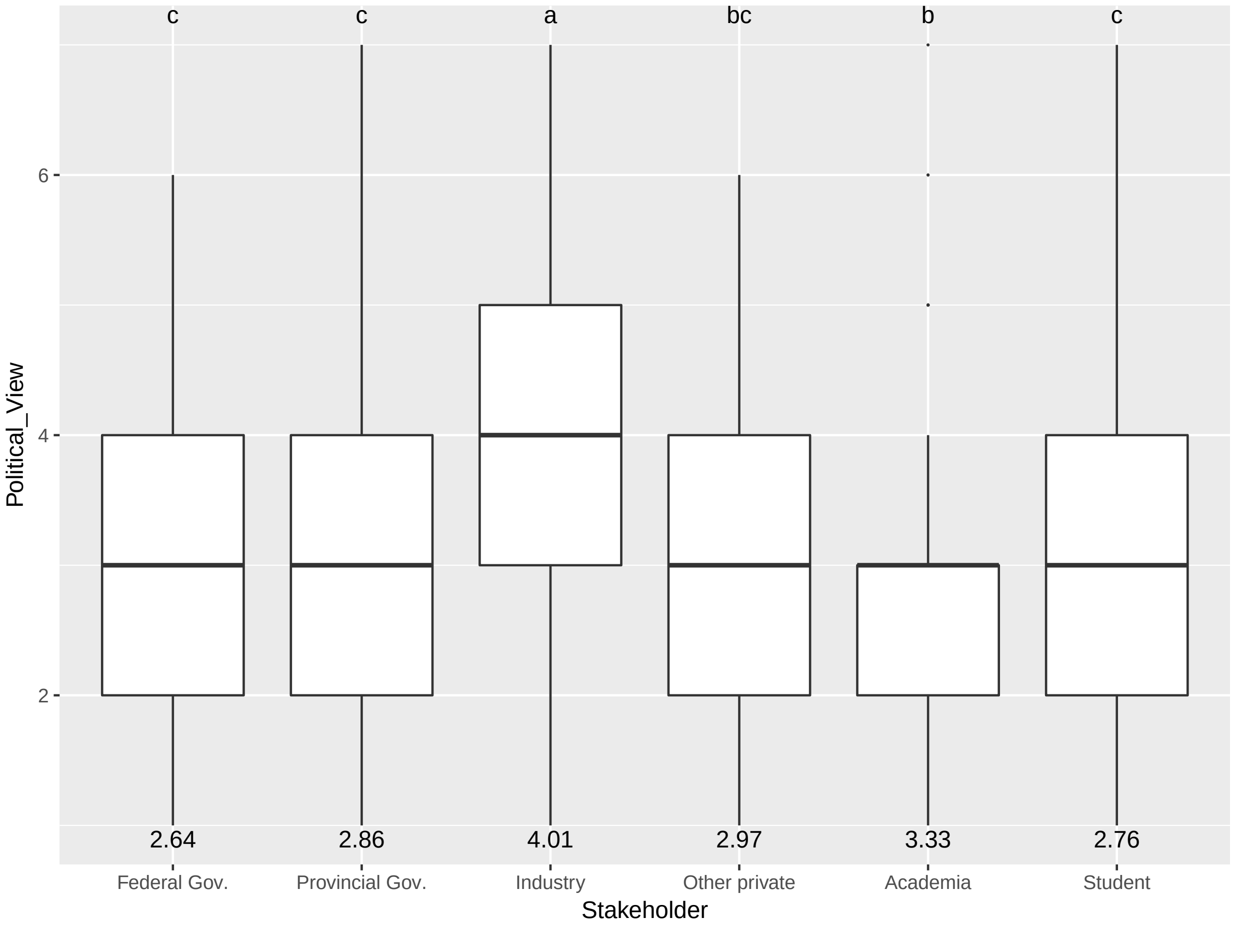

Figure S1.5. Distribution of political view of respondents in a survey on perceptions of climate change and its impacts on forest ecosystems in Canada as a function of stakeholder. Values below the boxplots are the mean political view for the stakeholder in a scale ranging from 1 (very liberal) to 7 (very conservative). Different letters above the boxplot indicate significant differences in the political view for those stakeholders For all boxplots, the central black line indicates the median value, lower and upper hinges indicate the first and third quartile and lower and upper limit of the vertical lines encompass the 95% interval.
