# Supplementary material for: Perceptions of climate change across the Canadian forest sector: The key factors of institutional and geographical environment
Source: PLoS One. 2018 Jun 13;13(6):e0197689. doi: 10.1371/journal.pone.0197689 (PMC5999070; doi:10.1371/journal.pone.0197689)
Supplement: S2 File — Responses by type of stakeholder for the first three sections of a survey on perceptions of climate change in the Canadian forest sector. (PDF) [file pone.0197689.s002.pdf]

Supplementary Material S2
Survey responses by type of stakeholder

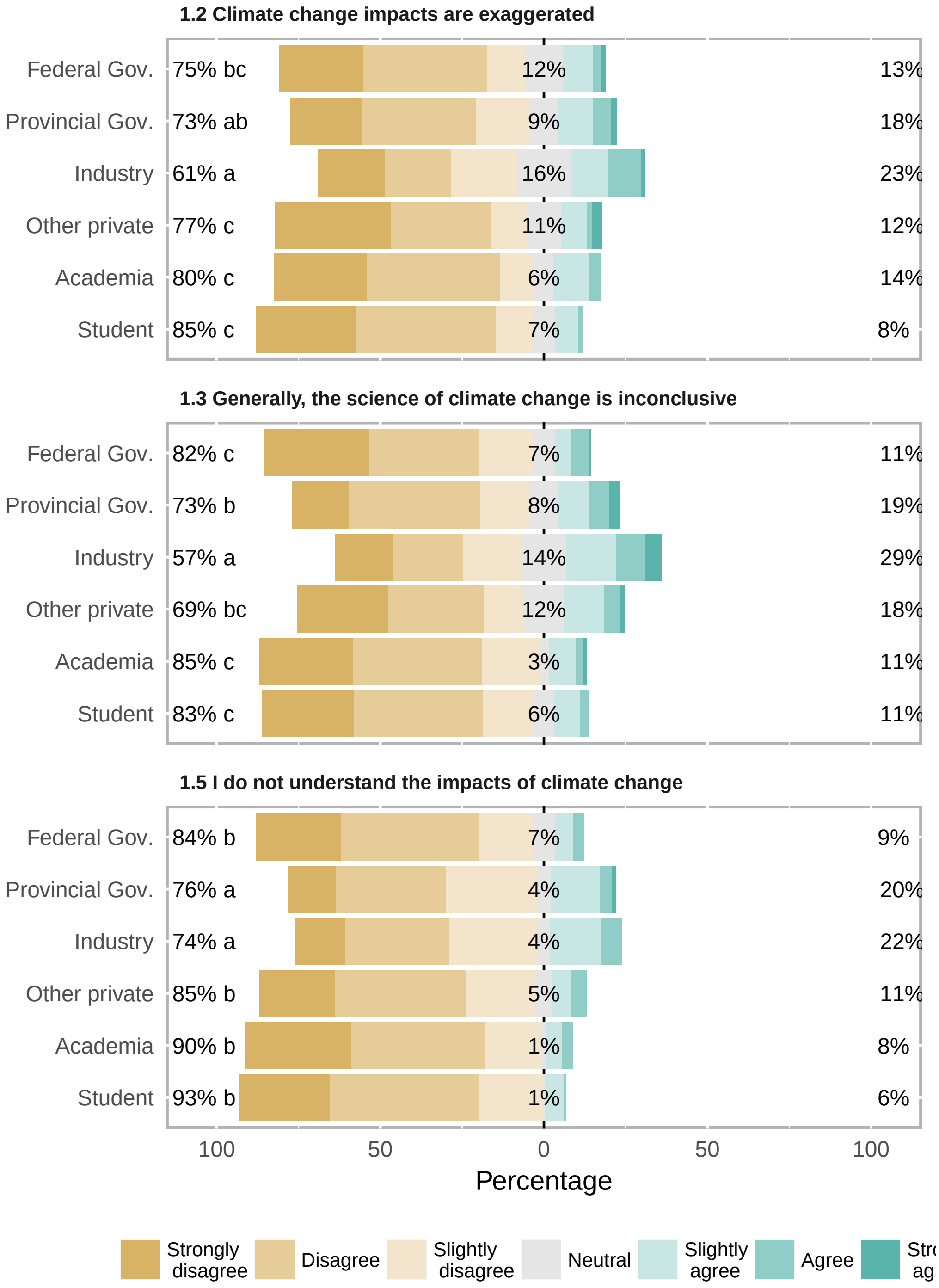

Figure S2.1 Responses by type of stakeholder for the first section of a survey conducted to capture perceptions on climate change and its impacts across the Canadian forest sector. The first section asked respondents about the general impacts of climate change. Percentages for each statement represent, from left to right, the amount of disagreements (strongly disagree, disagree, slightly disagree), neutral responses, and agreements (slightly agree, agree, strongly agree). Different letters beside the bars indicate significant differences in the distribution of responses across stakeholder types after a Kruskal-Wallis non-parametric test (n= 974).

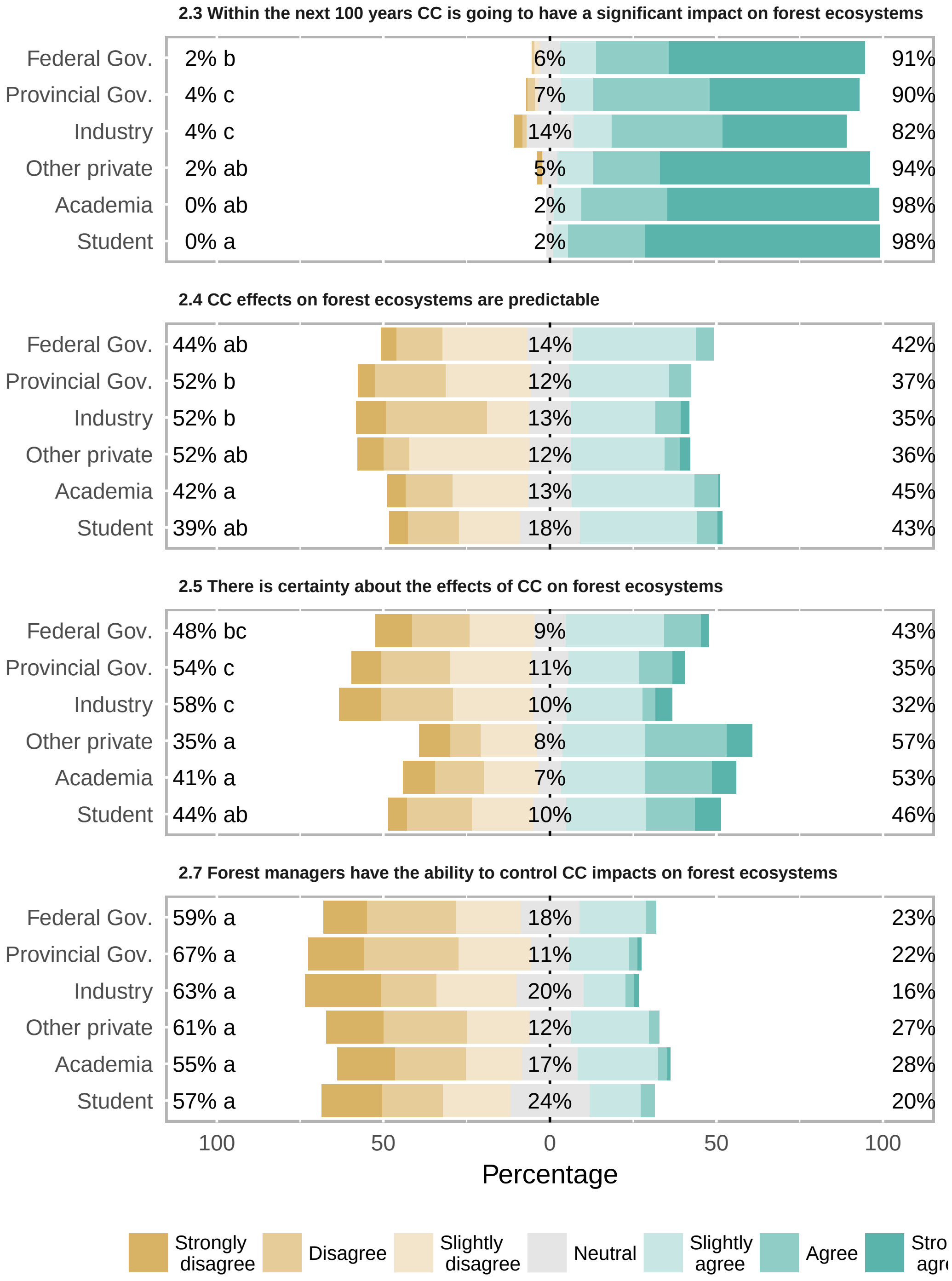

Figure S2.2 Responses by organization for the second section of a survey conducted to capture perceptions on climate change and its impacts across the Canadian forest sector. The second section asked respondents about the impacts of climate change on forest ecosystems. Percentages for each statement represent, from left to right, the amount of disagreements (strongly disagree, disagree, slightly disagree), neutral responses, and agreements (slightly agree, agree, strongly agree). Different letters beside the bars indicate significant differences in the distribution of responses across stakeholder types after a Kruskal-Wallis non-parametric test (n= 974).

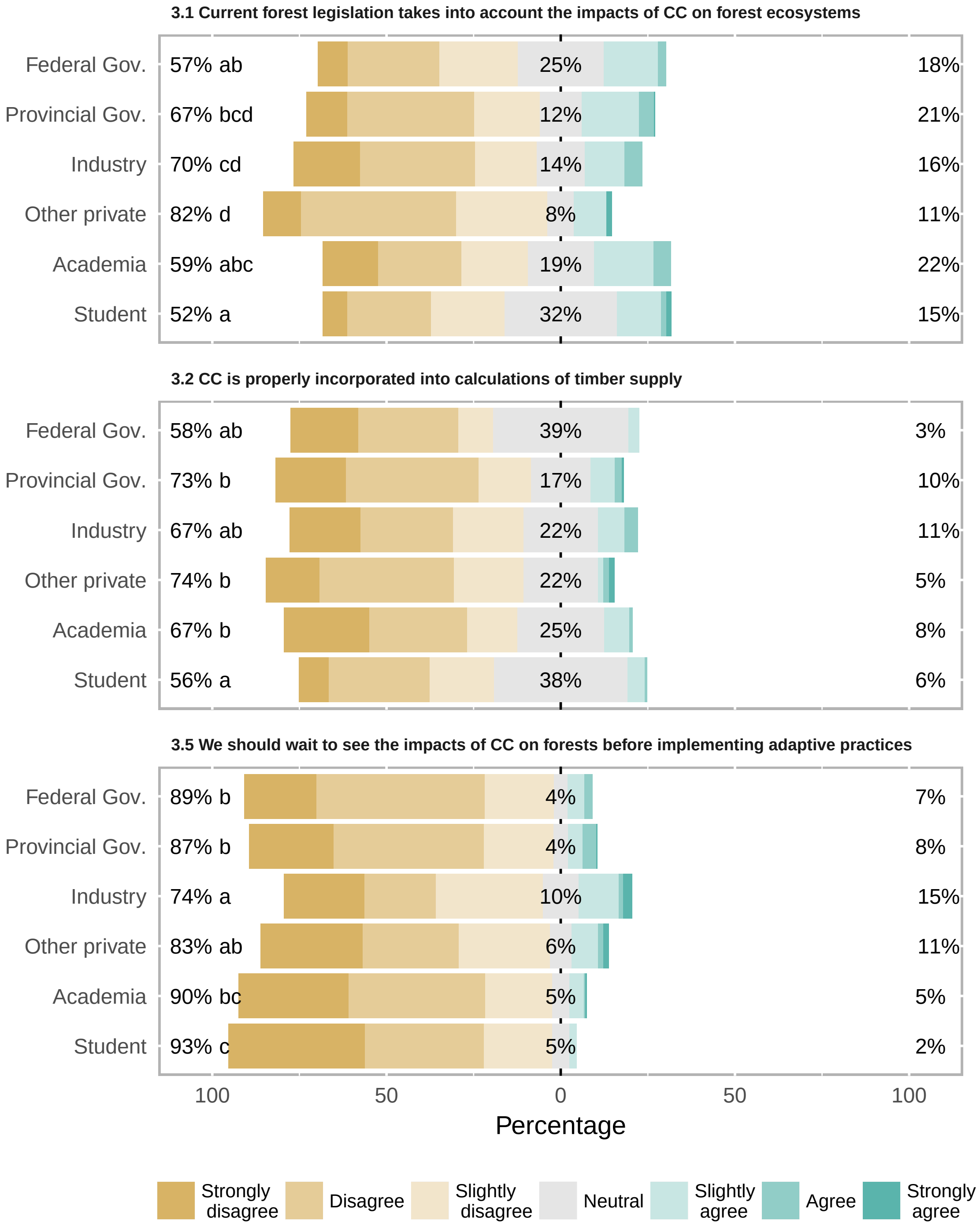

Figure S2.3 Responses by organization for the third section of a survey conducted to capture perceptions on climate change and its impacts across the Canadian forest sector. The third section asked respondents about the suitability of current forestry practices to face the impacts of climate change. Percentages for each statement represent, from left to right, the amount of disagreements (strongly disagree, disagree, slightly disagree), neutral responses, and agreements (slightly agree, agree, strongly agree). Different letters beside the bars indicate significant differences in the distribution of responses across stakeholder types after a Kruskal-Wallis non-parametric test (n= 974).
