# Supplementary material for: Perceptions of climate change across the Canadian forest sector: The key factors of institutional and geographical environment
Source: PLoS One. 2018 Jun 13;13(6):e0197689. doi: 10.1371/journal.pone.0197689 (PMC5999070; doi:10.1371/journal.pone.0197689)

Supplementary Material S4
Conditional Inference Trees

Conditional Inference (CI) classification trees for predicting perceptions of climate change and its impacts on forest ecosystems across the Canadian forest sector. Conditional Inference (CI) classification trees split the dataset into different groups based on certain values of the explanatory variables. At each intermediate mode, dataset is split into two groups based on the values of the explanatory variable indicated in the branches under the node. Stacked bar plot at each terminal node indicates the proportion of respondents that disagreed (dark gray), agreed (light gray) or were unsure (gray) about the statement. Total sample size depends on the statement, and can be calculated adding the sample size of the terminal nodes (indicated by brackets). Each tree only shows statistically significant variables at $p < 0.05$. The classification accuracy for each conditional inference tree is also provided. Education level can have 4 levels: *Non Univ*: non universitary studies; *BSc*: bachelor of sciences, *MSc*: Master of Sciences, *PhD*: doctorate. Provinces are *BC*: British Columbia; *AB:* ALberta; *ON*: Ontario; *QC*: Québec; *NB*: New Brunswick. Stakeholders are: *F.Gov*: Federal Govewrnment; *P.Gov*: Provincial Government; *Indus*: Industry; *Priv.*: other private organizations; *Acad*: academia (professors, researchers); *Stud*: graduate students.

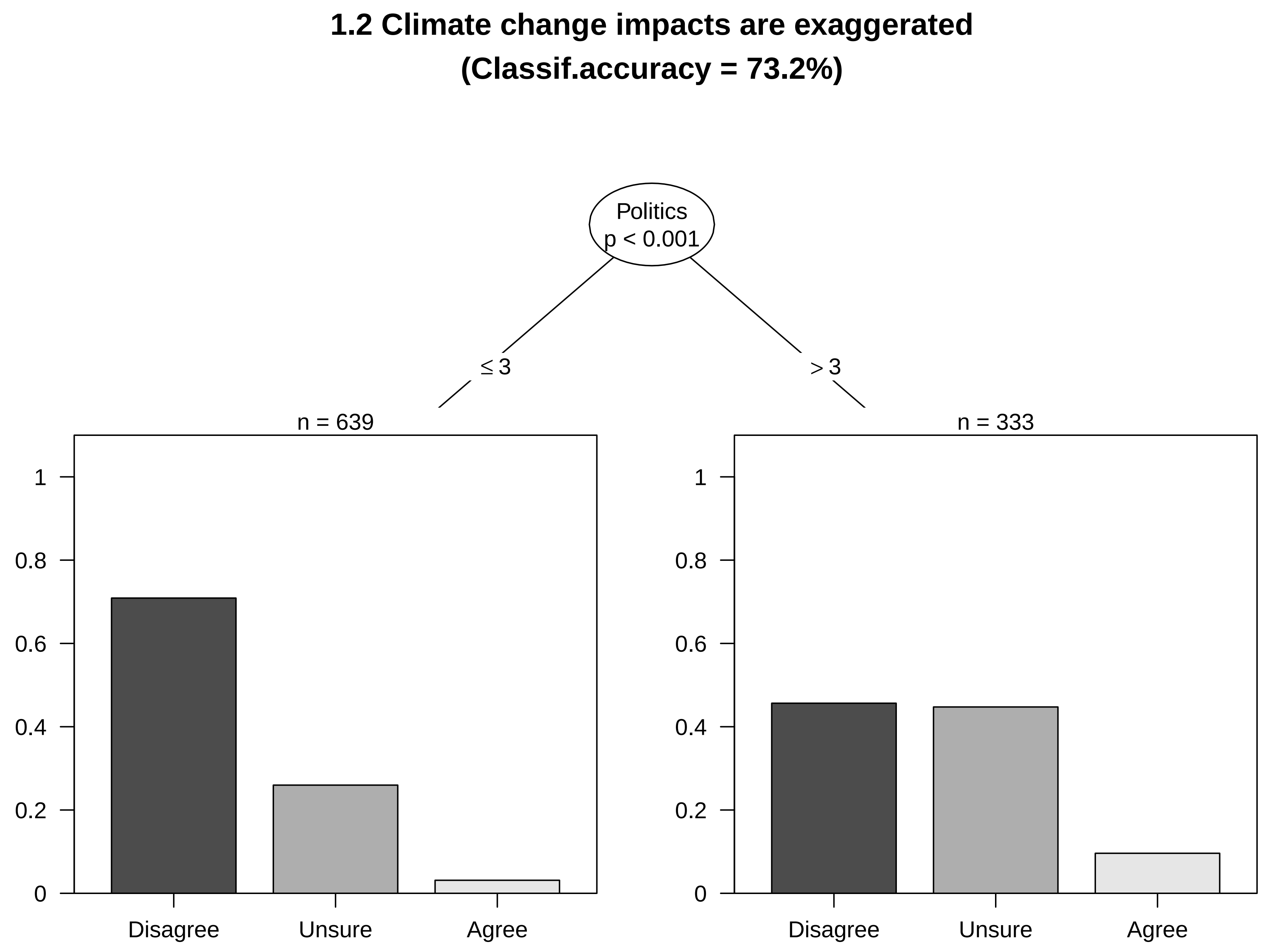
1.2 Climate change impacts are exaggerated
(Classif.accuracy = 73.2%)
Politics
p < 0.001
≤ 3
> 3
n = 639
n = 333
1
0.8
0.6
0.4
0.2
0
Disagree
Unsure
Agree

1.5 I do not understand the impacts of climate change
(Classif.accuracy = 76.7%)

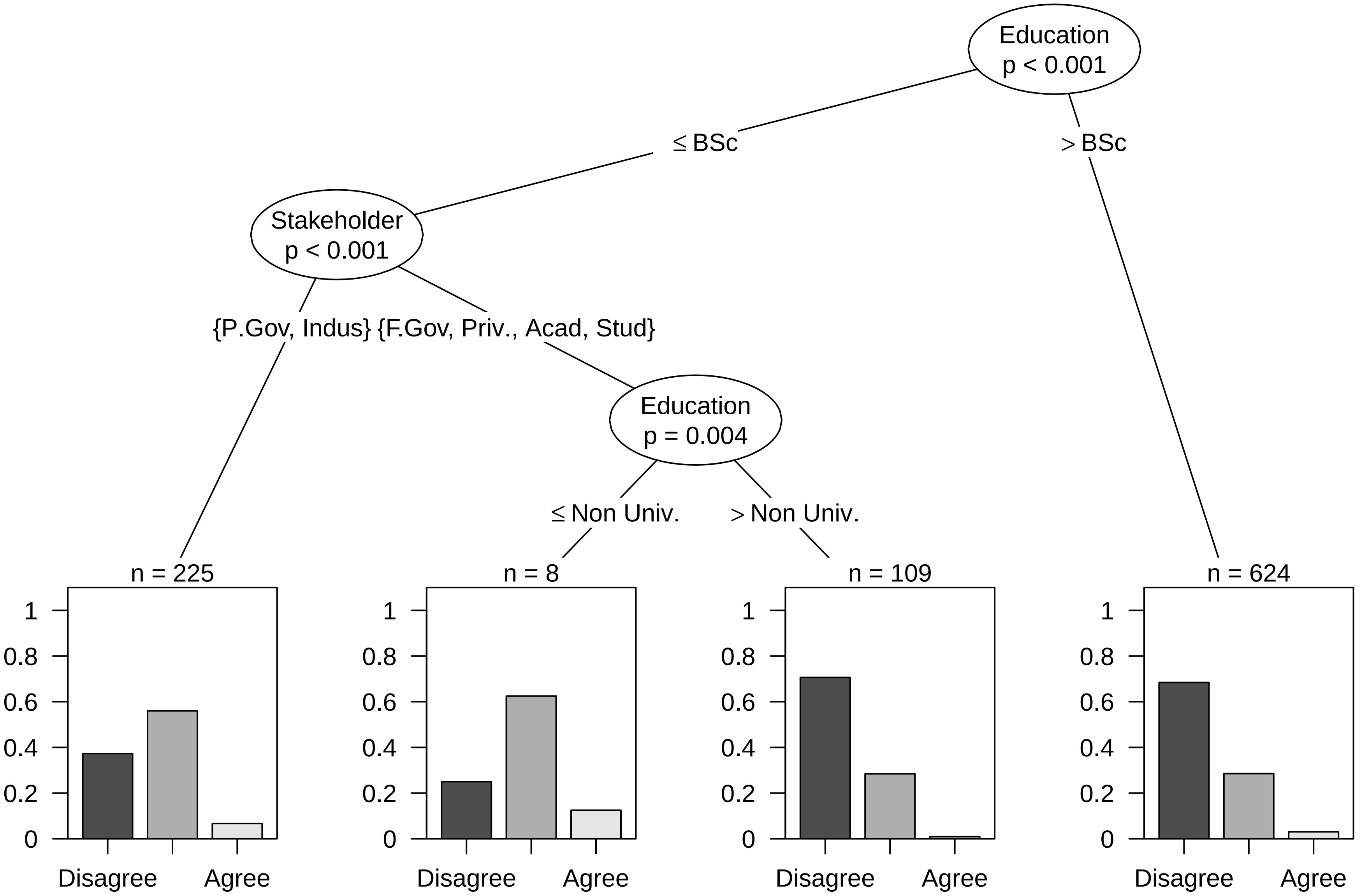

1.6 There is ample time to adapt to climate change
(Classif.accuracy = 71.1%)

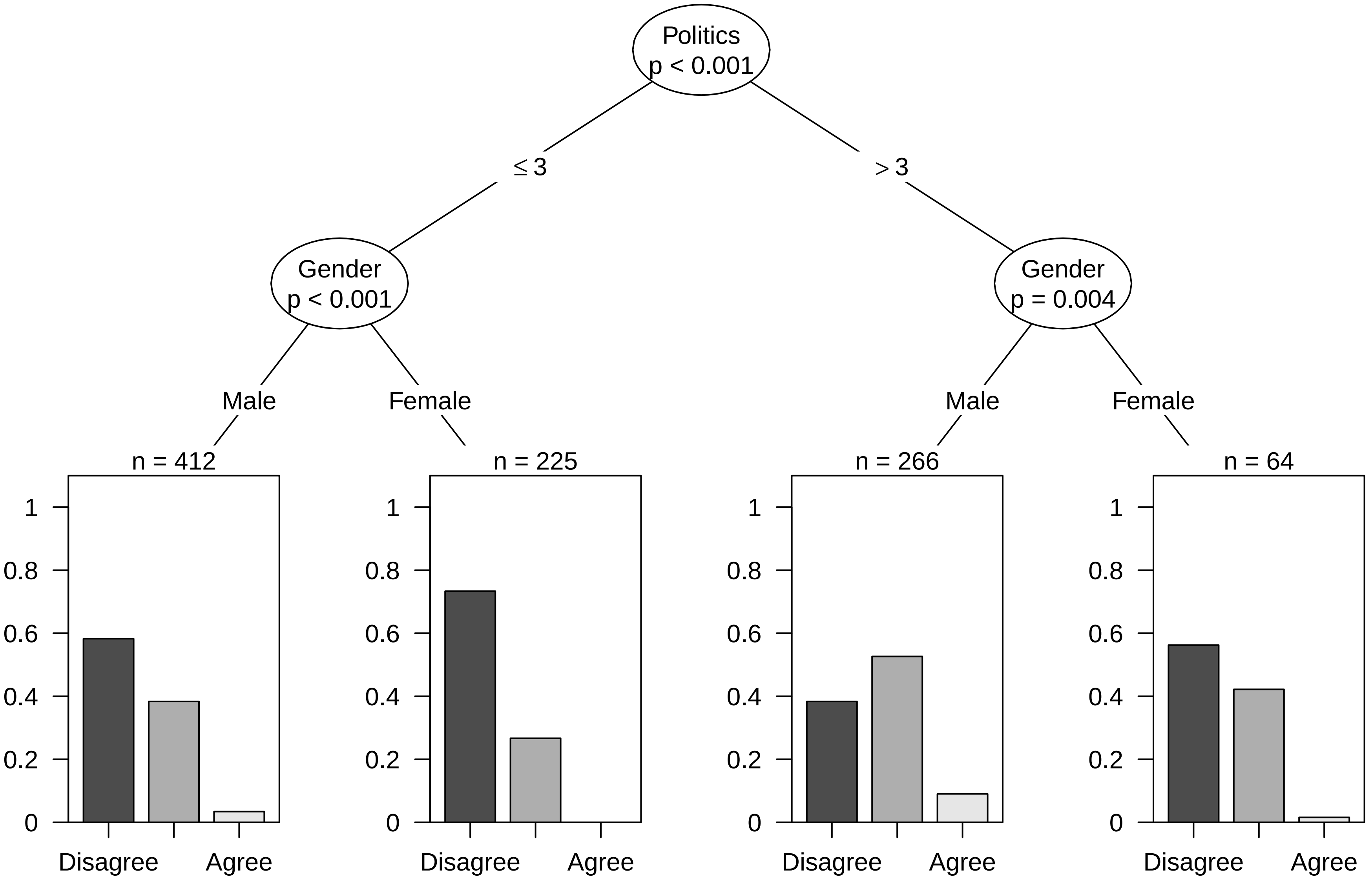

2.2 Within the next 50 years CC is going to have a significant impact on forest ecosystems (Classif.accuracy = 87.5%)

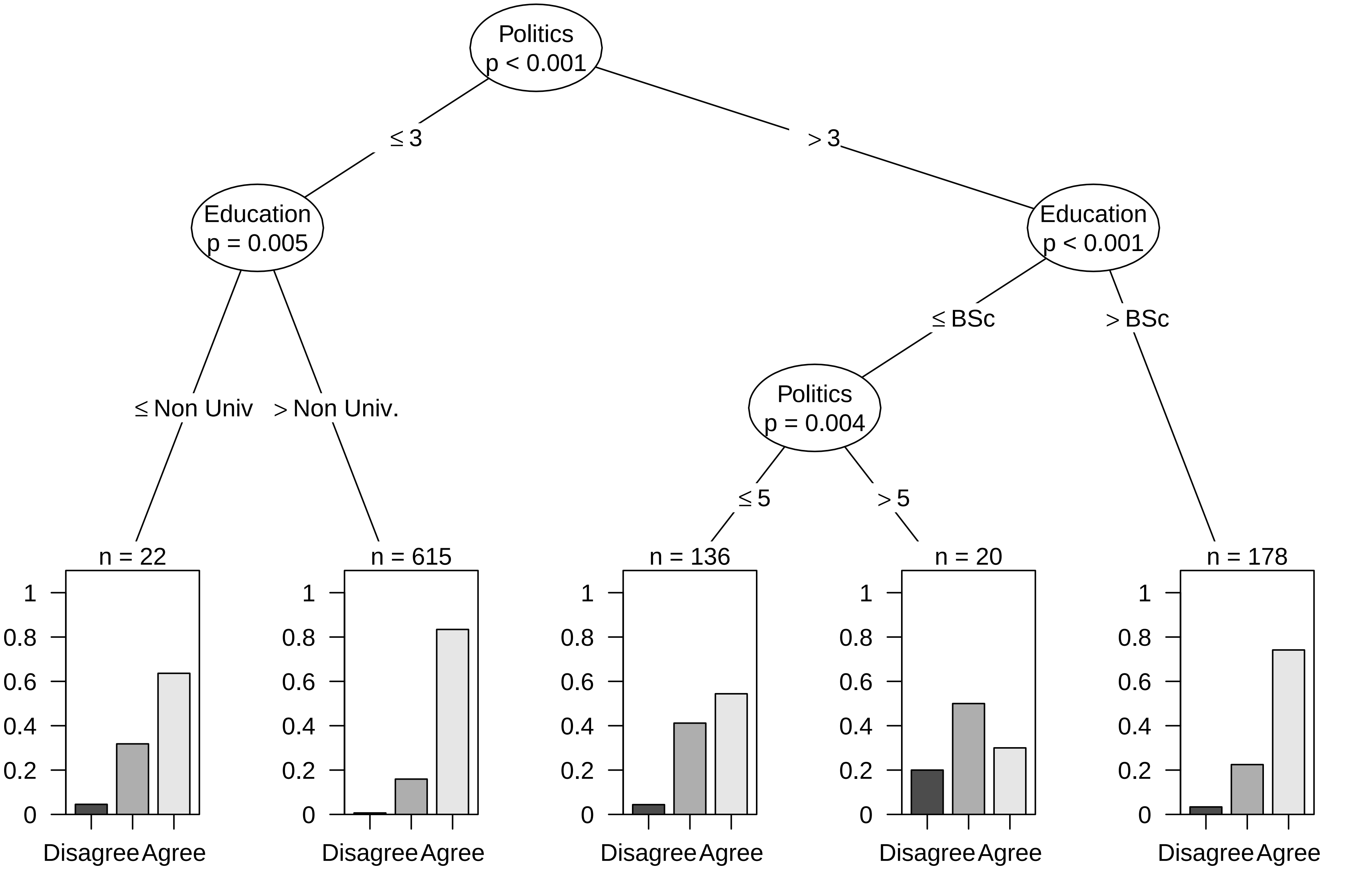

2.3 Within the next 100 years CC is going to have a significant impact on forest ecosystems (Classif.accuracy = 94.6%)

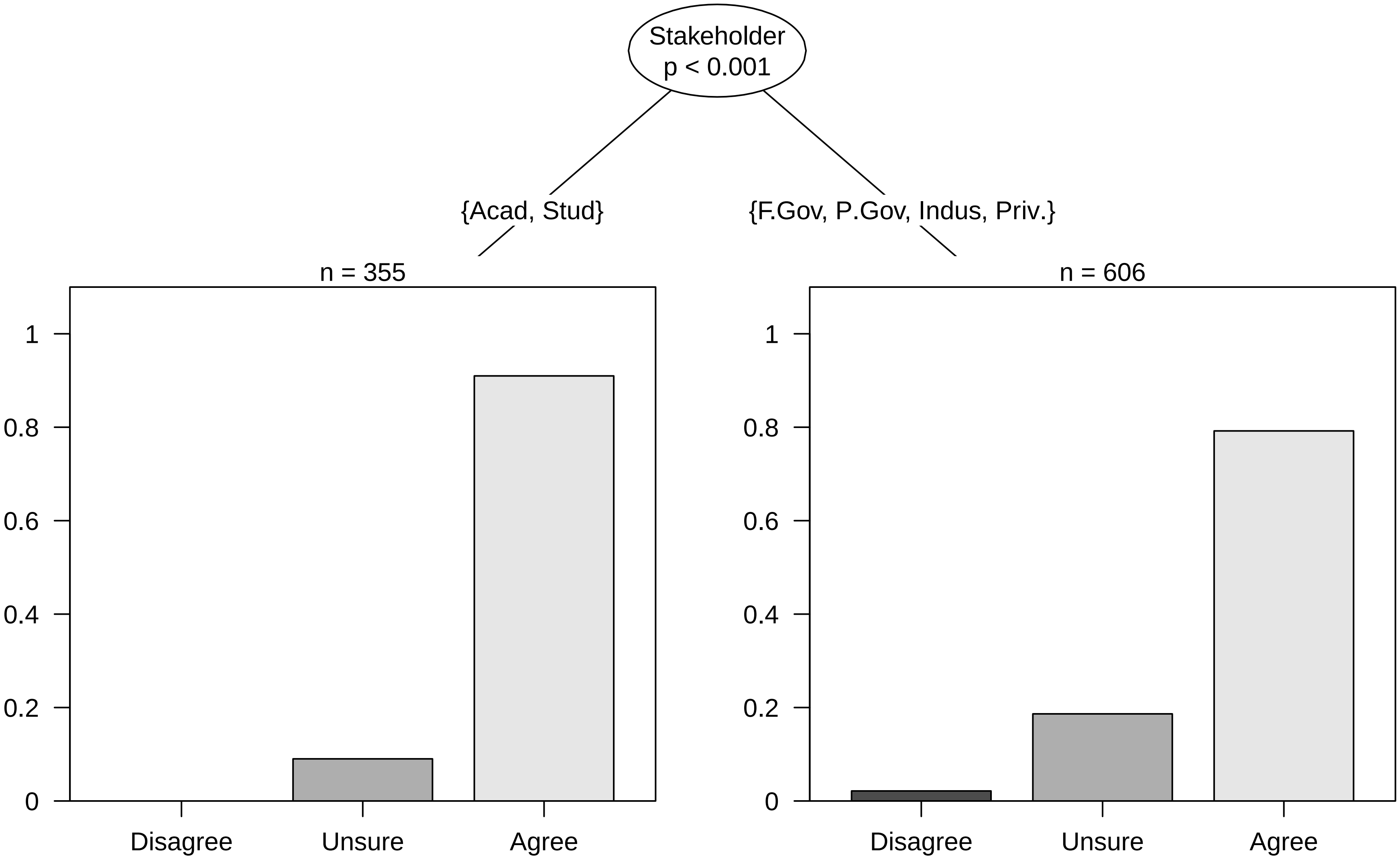

2.4 CC effects on forest ecosystems are predictable
(Classif.accuracy = 80.5%)

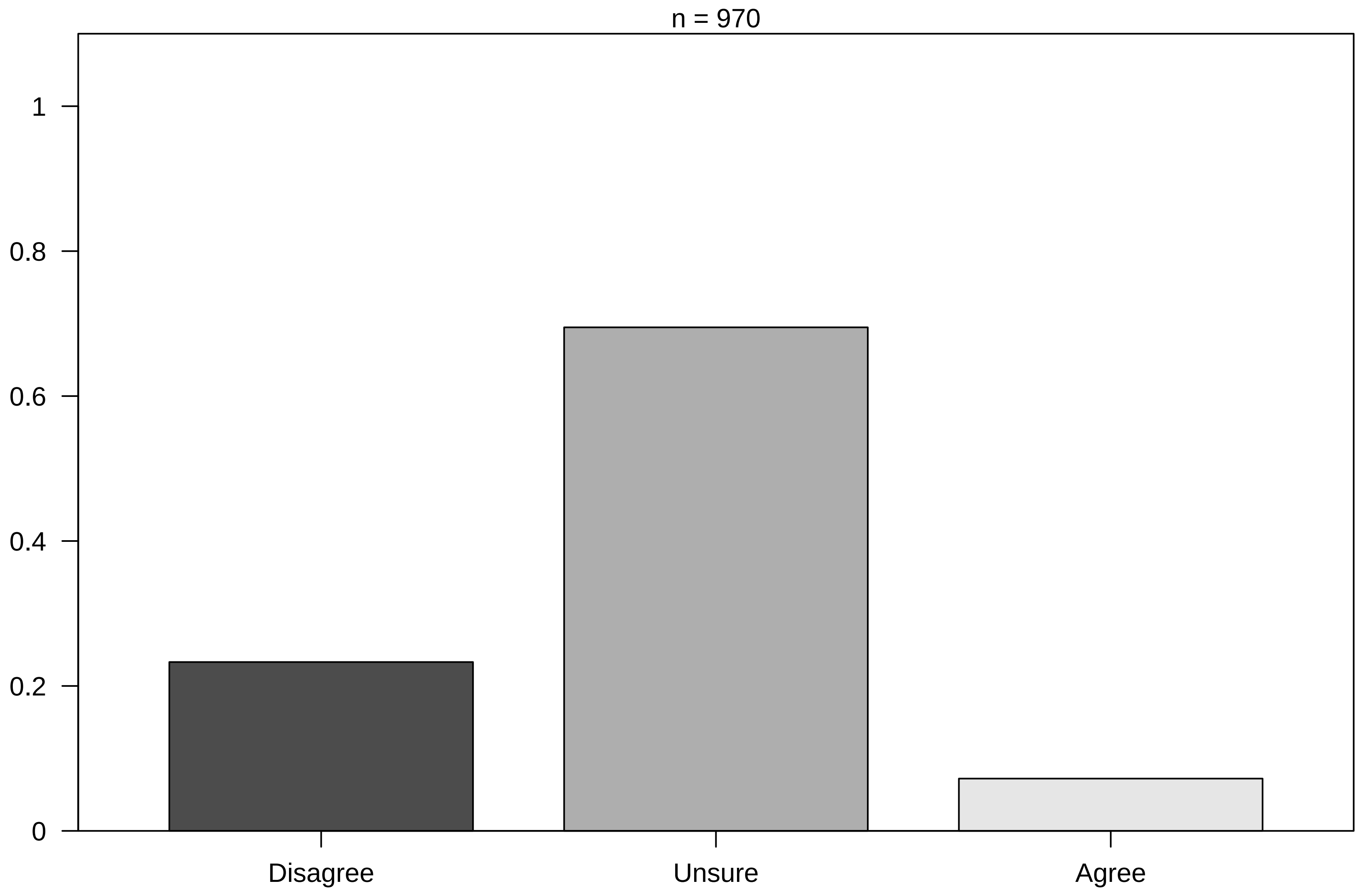

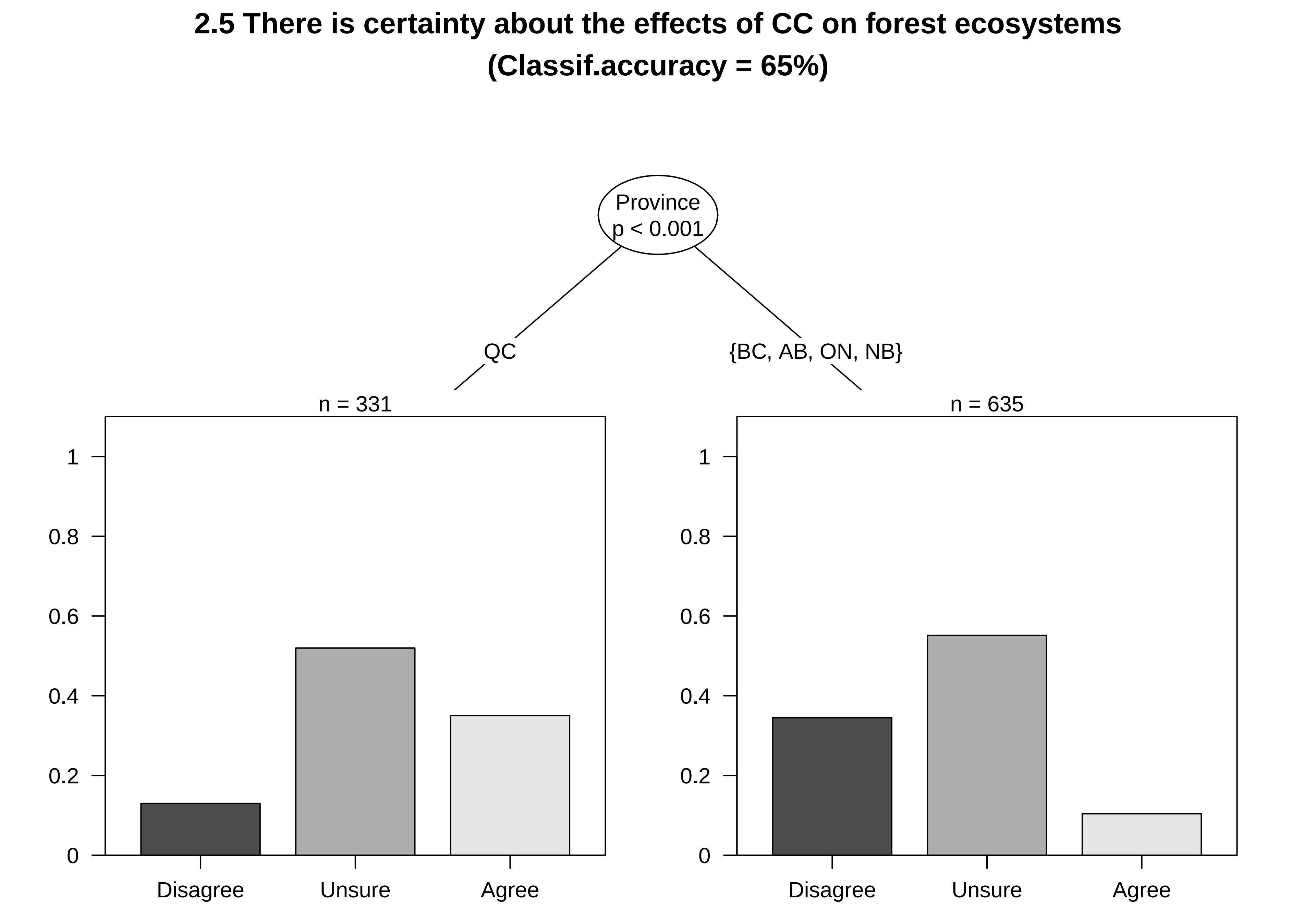
2.5 There is certainty about the effects of CC on forest ecosystems
(Classif.accuracy = 65%)
Province
p < 0.001
QC
{BC, AB, ON, NB}
n = 331
n = 635
1
0.8
0.6
0.4
0.2
0
Disagree
Unsure
Agree

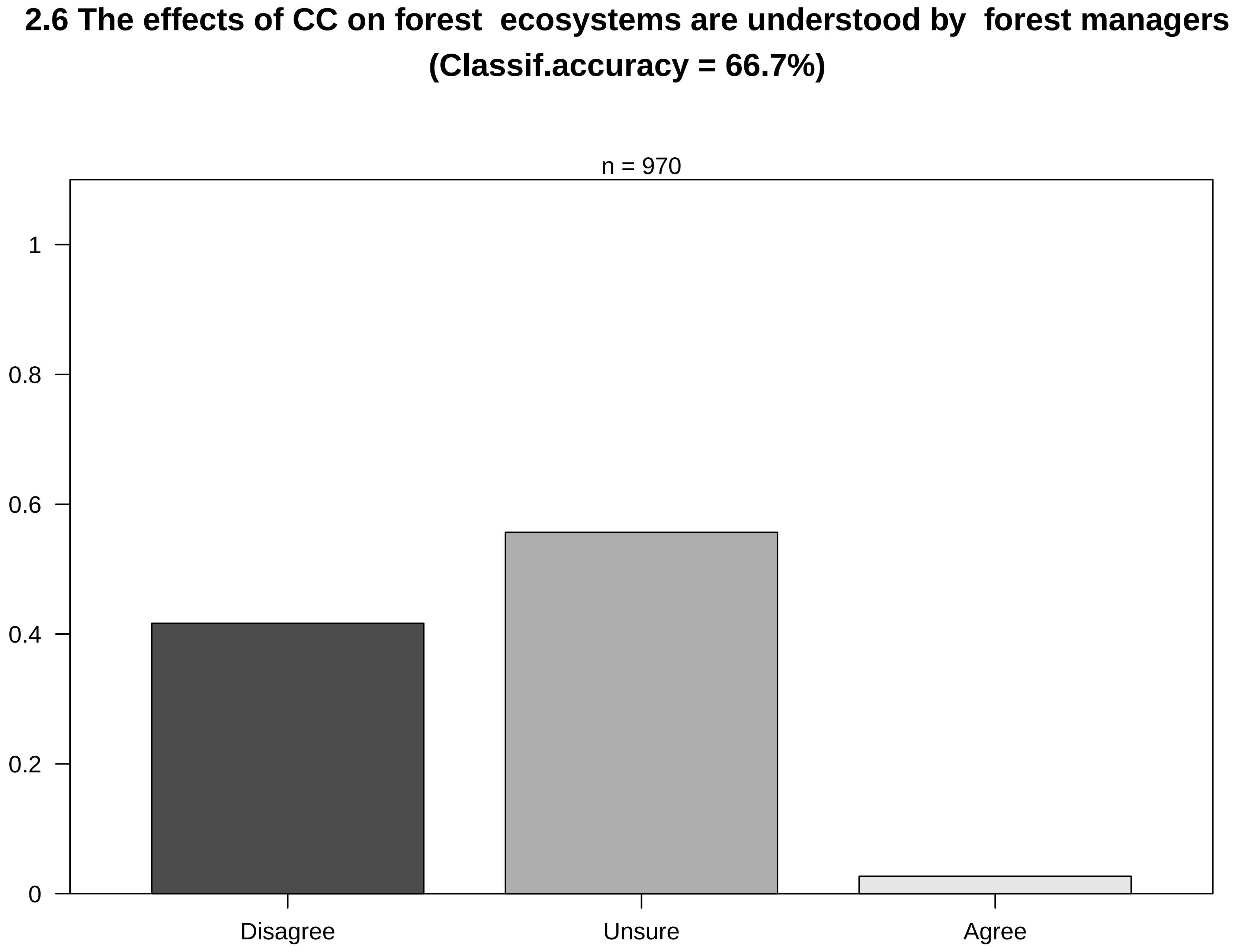
2.6 The effects of CC on forest ecosystems are understood by forest managers
(Classif.accuracy = 66.7%)
n = 970
1
0.8
0.6
0.4
0.2
0
Disagree
Unsure
Agree

2.7 Forest managers have the ability to control CC impacts on forest ecosystems (Classif.accuracy = 69.3%)

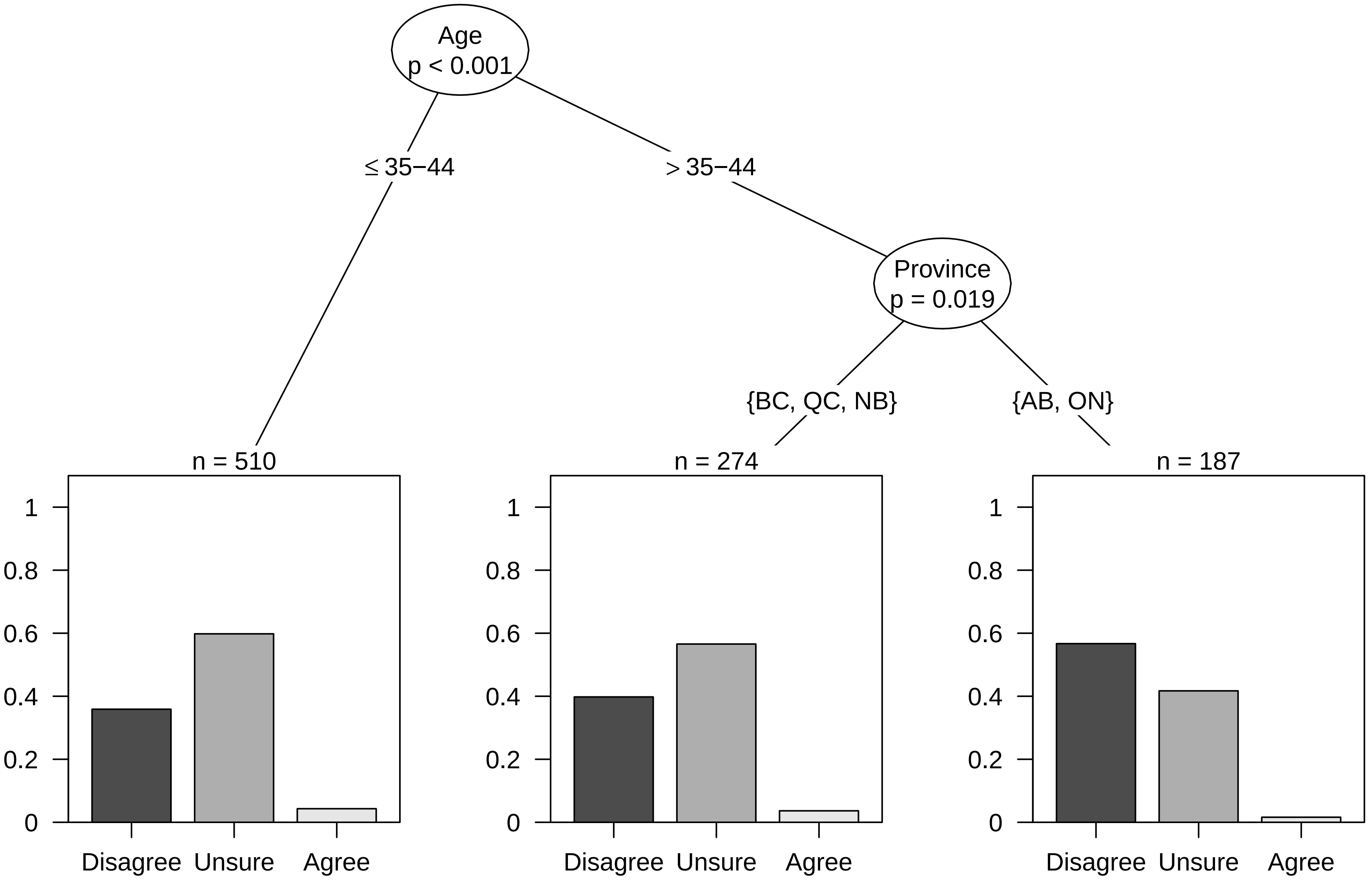

3.1 Current forest legislation takes into account the impacts of CC on forest ecosystems (Classif.accuracy = 67.6%)

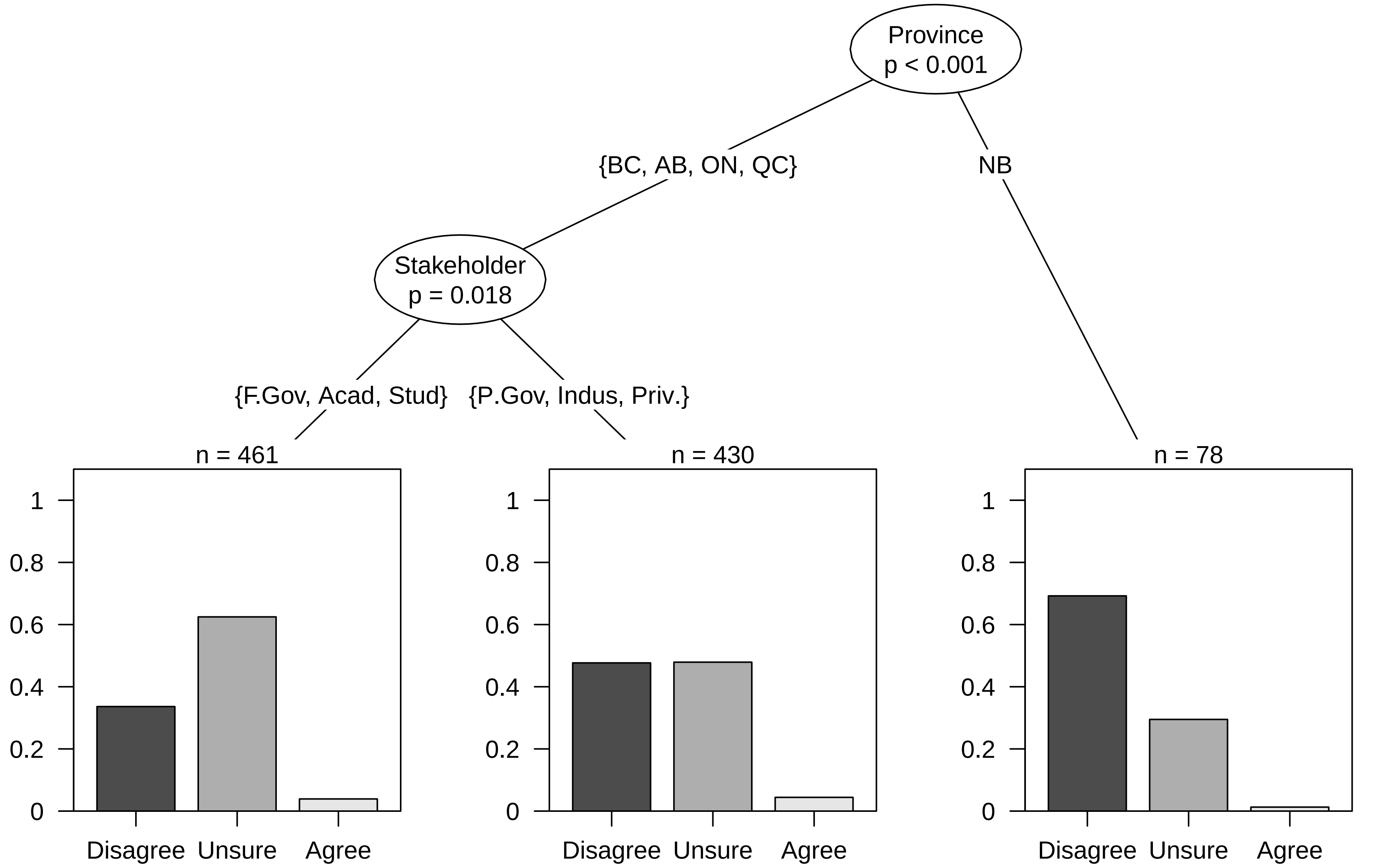

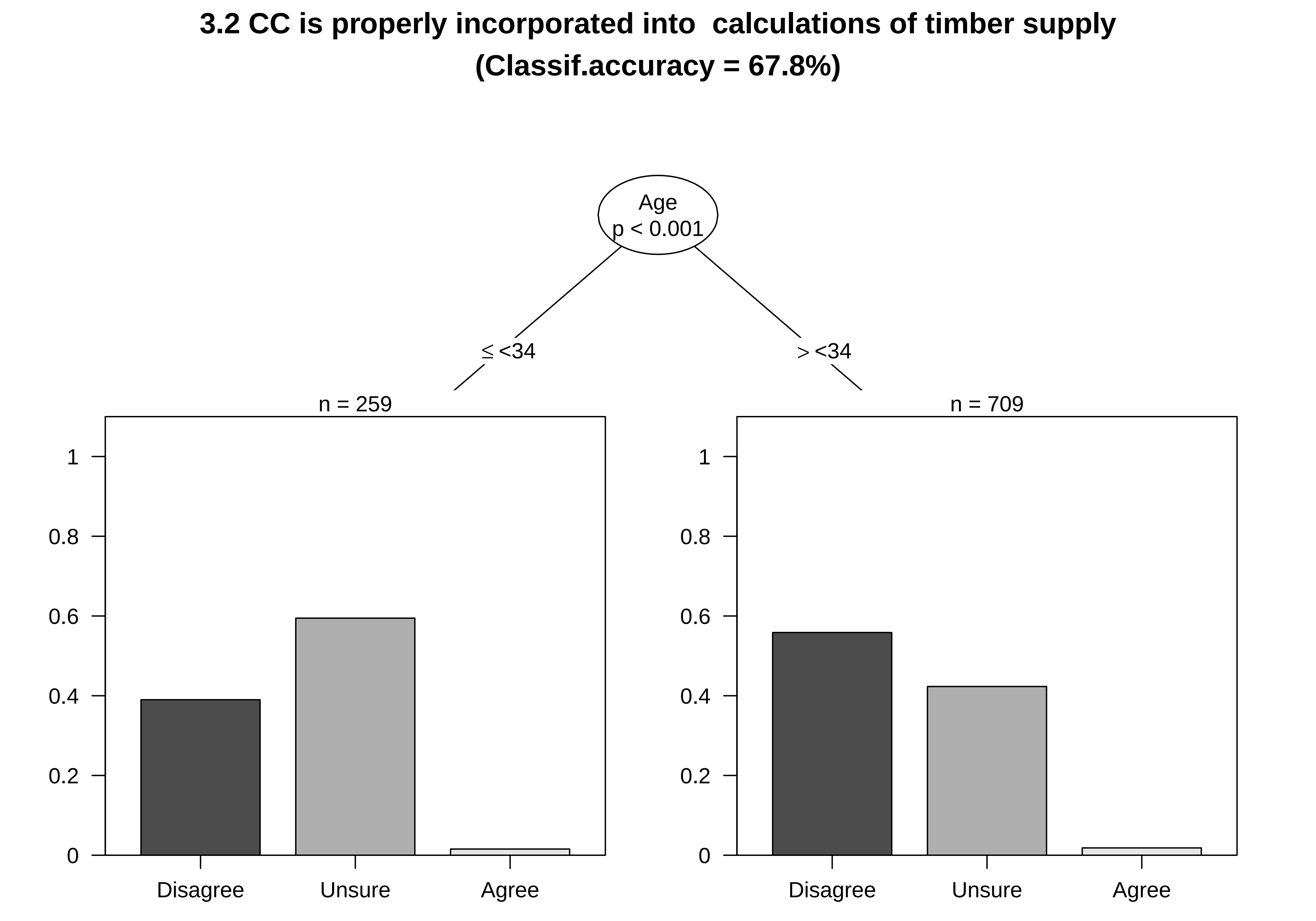
3.2 CC is properly incorporated into calculations of timber supply
(Classif.accuracy = 67.8%)
Age
p < 0.001
≤ <34
> <34
n = 259
n = 709
1
0.8
0.6
0.4
0.2
0
Disagree
Unsure
Agree

.3 The forest practices currently implemented are sufficient to face the impacts of CC on forest

(Classif.accuracy = 67%)

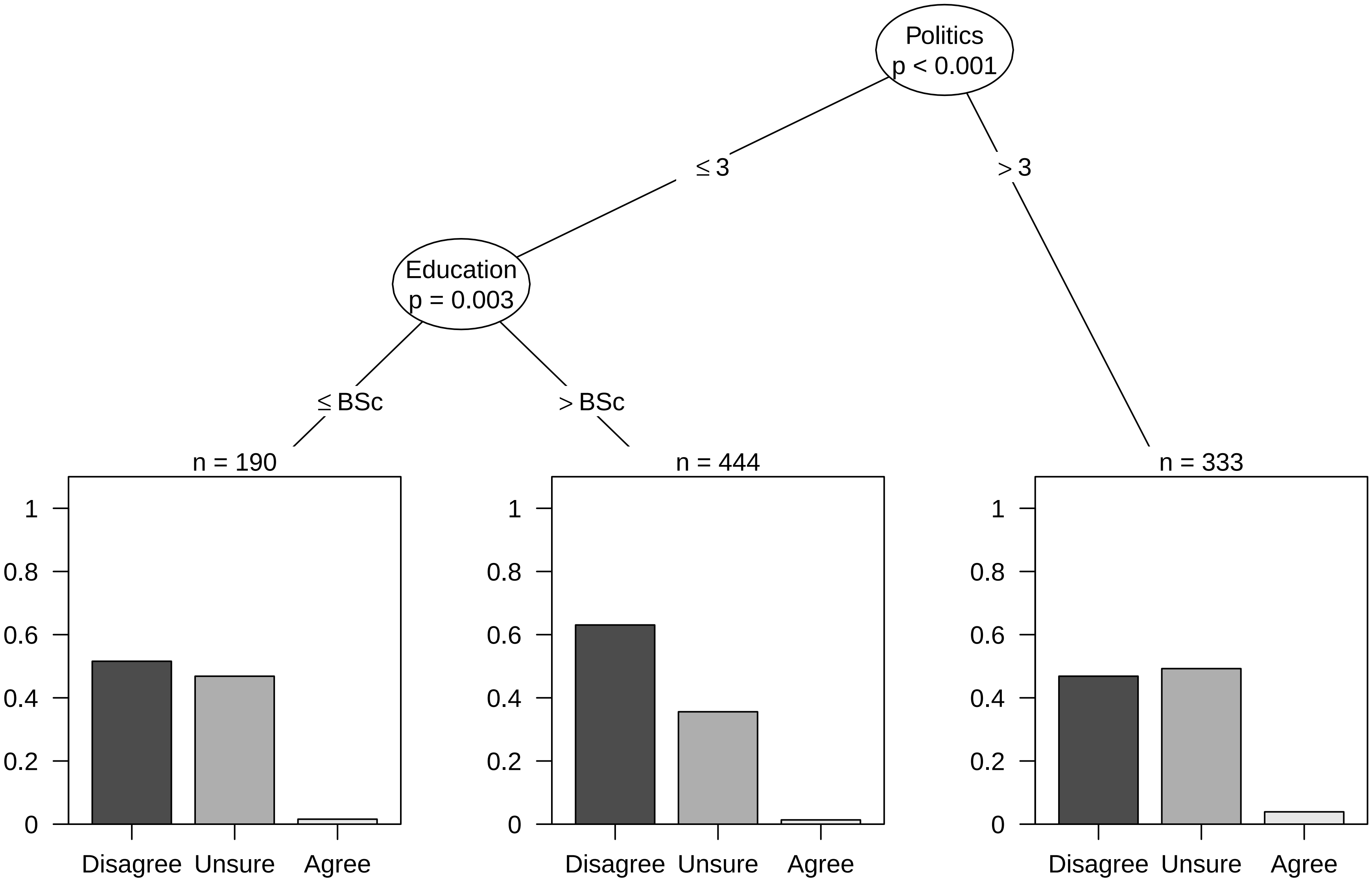

3.4 We need to create and design new forest practices to deal with the impacts of CC on forests

(Classif.accuracy = 74.9%)

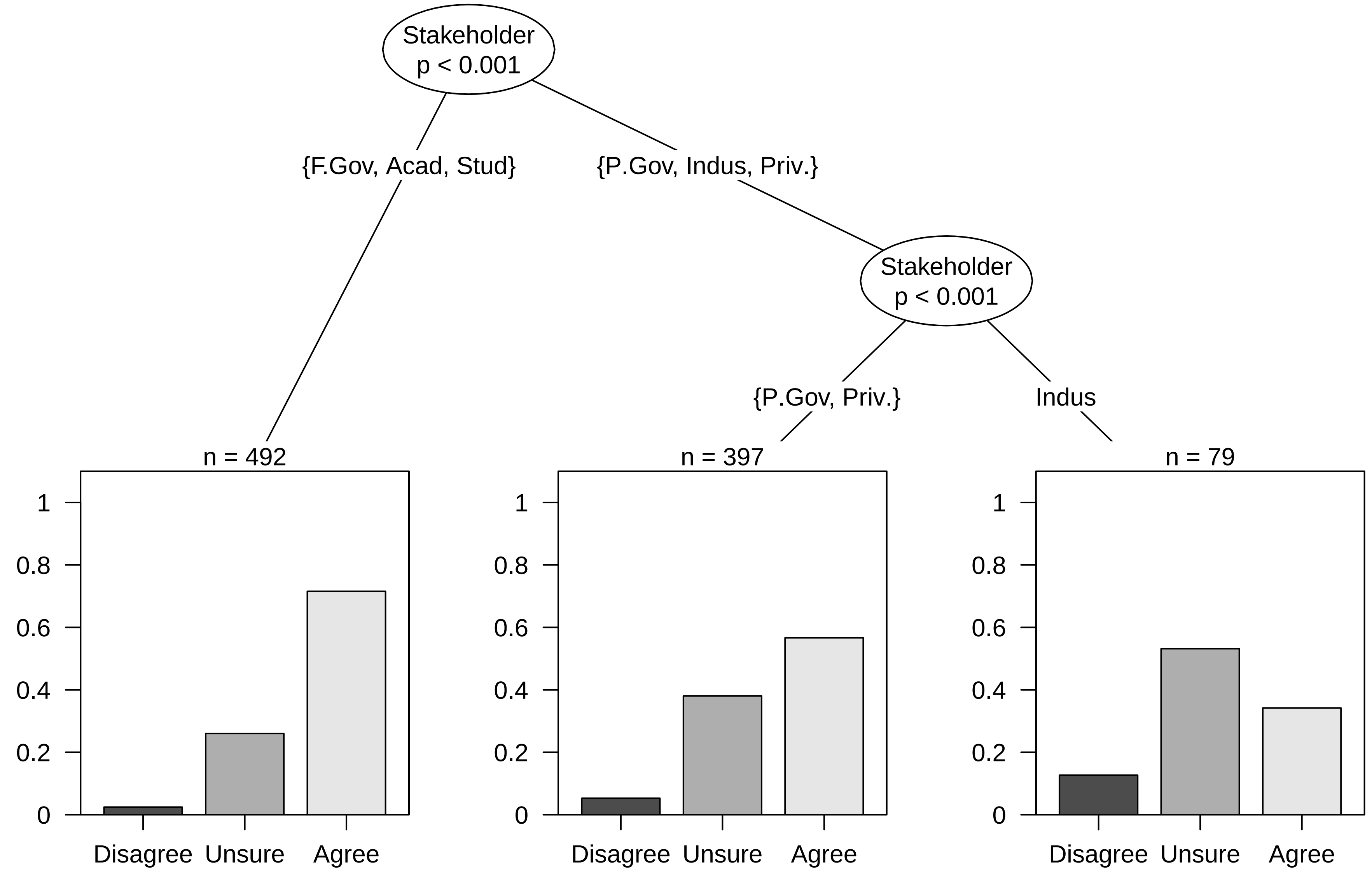

3.5 We should wait to see the impacts of CC on forests before implementing adaptive practices (Classif.accuracy = 77.7%)

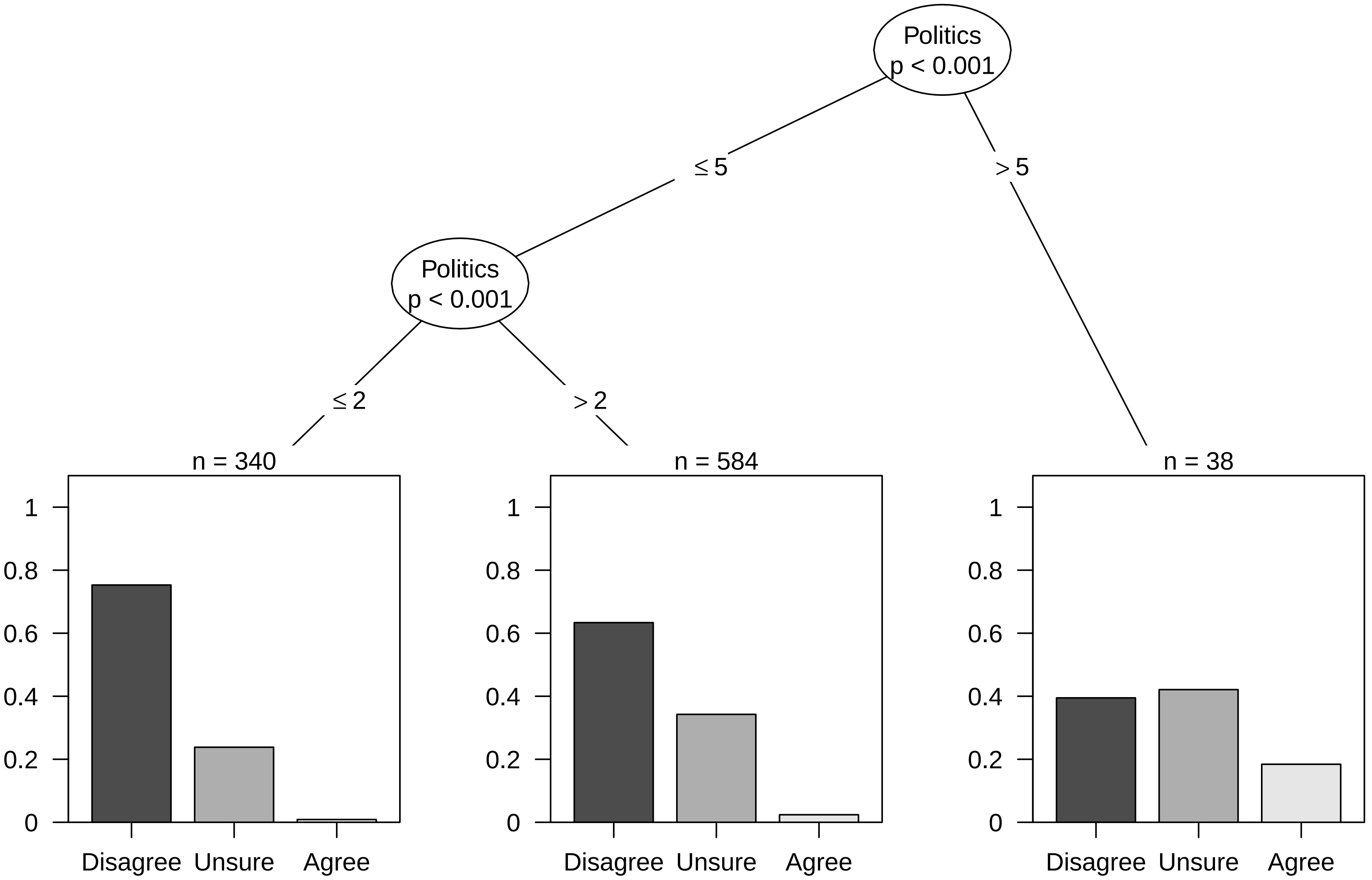

Supplement: S4 File — Conditional Inference (CI) classification trees for predicting perceptions of climate change and its impacts on forest ecosystems across the Canadian forest sector. (PDF) [file pone.0197689.s004.pdf]
